# Personality and Suicidal Behavior in Old Age: A Systematic Literature Review

**DOI:** 10.3389/fpsyt.2018.00128

**Published:** 2018-05-07

**Authors:** Anna Szücs, Katalin Szanto, Jean-Michel Aubry, Alexandre Y. Dombrovski

**Affiliations:** ^1^Department of Psychiatry, Faculty of Medicine, University of Geneva, Geneva, Switzerland; ^2^Decision Neuroscience and Psychopathology Laboratory, Department of Psychiatry, School of Medicine, University of Pittsburgh, Pittsburgh, PA, United States

**Keywords:** aged, elderly, suicide, attempted suicide, suicidal ideation, personality, personality disorder, Five-Factor Model

## Abstract

**Background:**

Suicide rates generally peak in the second half of life and are particularly high in older men; however, little is known about the contribution of dispositional factors to late-life suicide. Maladaptive personality traits have been strongly implicated in suicide among younger adults, but the extent to which they continue to play a role in late-life suicidal behavior is unclear. We also do not know whether specific personality profiles interact with the stressors of aging to cause suicidal behavior.

**Methods:**

We sought to synthesize the data on personality pathology in late-life suicidal ideation and behavior *via* a systematic review using the PubMed, Google Scholar, PsycInfo, Scopus, Ovid, Web of Science, Embase, and Cochrane search engines. The included key words related to three descriptors: “personality,” “suicide,” and “elderly.” Included articles evaluated personality based on the Five-Factor Model (FFM) or ICD/DSM diagnostic criteria in older samples with minimum age cutoffs of 50 years or older. Our original search identified 1,183 articles, of which 31 were retained.

**Results:**

Included studies were heterogeneous in their design and personality measurements. Studies of categorical personality disorders were particularly scarce and suggested a stronger association with late-life suicidal ideation than with death by suicide. Only obsessive–compulsive and avoidant personality traits were associated with death by suicide in old age, but only in studies that did not control for depression. All personality constructs were positively linked to suicidal ideation, except for histrionic personality, which emerged as a negative predictor. Studies employing the FFM also indicated that older adults who died by suicide were less likely to display a maladaptive personality profile than elderly suicide attempters and younger suicide victims, having both lower levels of neuroticism and higher levels of conscientiousness than these comparison groups. Nevertheless, older suicide victims displayed lower levels of openness to experience than younger victims in two samples.

**Conclusion:**

Maladaptive personality manifests in milder, subthreshold, and more heterogeneous forms in late-life vs. early-life suicide. An inability to adapt to the changes occurring in late life may help explain the association between suicide in old age and higher conscientiousness as well as obsessive–compulsive and avoidant personality disorders.

## Introduction

### Rationale

There has been a continuous rise in suicide rates in the United States, increasing from 10.5 to 13 per 100,000 between 1999 and 2014 (1). Suicide rates peak after age 45 for both males and females, with the highest rates in the population found in older white men aged 85 or older, for whom rates are as high as 50.7 per 100,000 (2). The known risk factors for suicide in old age remain largely unspecific, however, giving us only a limited understanding of the psychological mechanisms involved. For example, depression, physical decline, family discord, social isolation, and financial issues are well-established risk factors for suicide (3–6), but they are also present in many older adults who never engage in suicidal behavior. According to the stress–diathesis model, suicidal individuals may possess lifelong traits of vulnerability, and it is the interaction between such traits and acute stressors that triggers suicidal behavior (7). Maladaptive personality traits are strongly implicated both with suicide among younger adults and with failure to cope with the stressors of aging (8–10). Thus, the stress–diathesis framework informs two questions. First, to what extent do the personality features implicated in suicide among younger adults confer suicide risk in old age? Second, what personality profiles are uniquely associated with late-life suicide by virtue of their interaction with the stressors of aging (e.g., physical decline)? We can even entertain the possibility of pleiotropic effects (11, 12) wherein traits that confer a reproductive advantage earlier in life could increase suicide risk in old age. To the best of our knowledge, in the existing literature, there is no consensus on these questions.

On the other hand, an association between personality disorders and suicidal behavior has been reported in most studies examining younger adults (13, 14). All personality disorders, with the exception of schizoid and histrionic, are associated with increased suicide risk in clinical or community-dwelling adult samples (14–18). Studies using dimensional models of personality also link both suicide attempts and death by suicide with high neuroticism and low extraversion, two traits that are also correlated with depression and broadly defined psychopathology (19–23). Borderline personality disorder (BPD) has been most strongly implicated in adult suicidal behavior, being linked to multiple attempts starting at an earlier age, and often mediated by impulsivity (14, 24, 25). However, externalizing aspects of BPD and impulsive traits specifically are most severe in young adulthood and tend to subside with age (26, 27). Moreover, in contrast to the high-impulsivity, lower-lethality suicidal behavior of younger age groups, the elderly tend to carry out fewer but higher-lethality suicidal acts, characterized by careful planning, and often occurring without warning signs (28, 29). These observations suggest that suicidal behavior in old age may be associated with personality profiles that are distinct from borderline and Cluster B pathology.

### Objectives

This study aims to provide a comprehensive overview of the existing literature on the personality profiles of the suicidal elderly. Section “Results” first summarizes the characteristics of the studies, then reports data on personality pathology as a whole, and finally focuses separately on each DSM personality disorder and Five-Factor domain. Although the definition of personality can encompass *all individual differences in characteristic patterns of thinking, feeling, and behaving* (30), our theoretical focus on categorical personality disorders and the Big Five precluded us from reviewing studies that assessed other trait-like constructs, such as impulsivity or aggression. In this study, we considered behavior to be suicidal if it was self-injurious and enacted with intent to kill oneself, and suicidal ideation as thoughts of taking one’s own life (31). These definitions do not include para-suicide or passive death wish. Even though ideation, attempt, and death by suicide are interconnected entities, they do not correspond to entirely identical clinical populations, since the majority of ideators will never attempt suicide (32), and the majority of attempters do not die by suicide (33). Supporting this view, distinct yet partially overlapping personality profiles have been found in adult suicide ideators and attempters (34–36). Thus, this review will address findings on death by suicide, attempted suicide, and suicidal ideation separately in each subsection.

### Research Question

The question we sought to answer is whether elderly who engage in suicidal behavior display qualitatively different personality profiles compared to their younger counterparts. Given the abatement of externalizing behaviors with aging, including BPD symptoms, we hypothesized that the contribution of these traits to suicidal behavior will diminish in old age. Furthermore, we sought to clarify whether differences in personality profiles exist in the elderly between those with ideation exclusively, attempt, and death by suicide. Based on the findings in younger samples mentioned earlier, we expect to see an overall stronger association of dysfunctional personality with suicidal ideation than death by suicide, and moderate levels associated with attempted suicide.

## Methods

**Figure 1:**
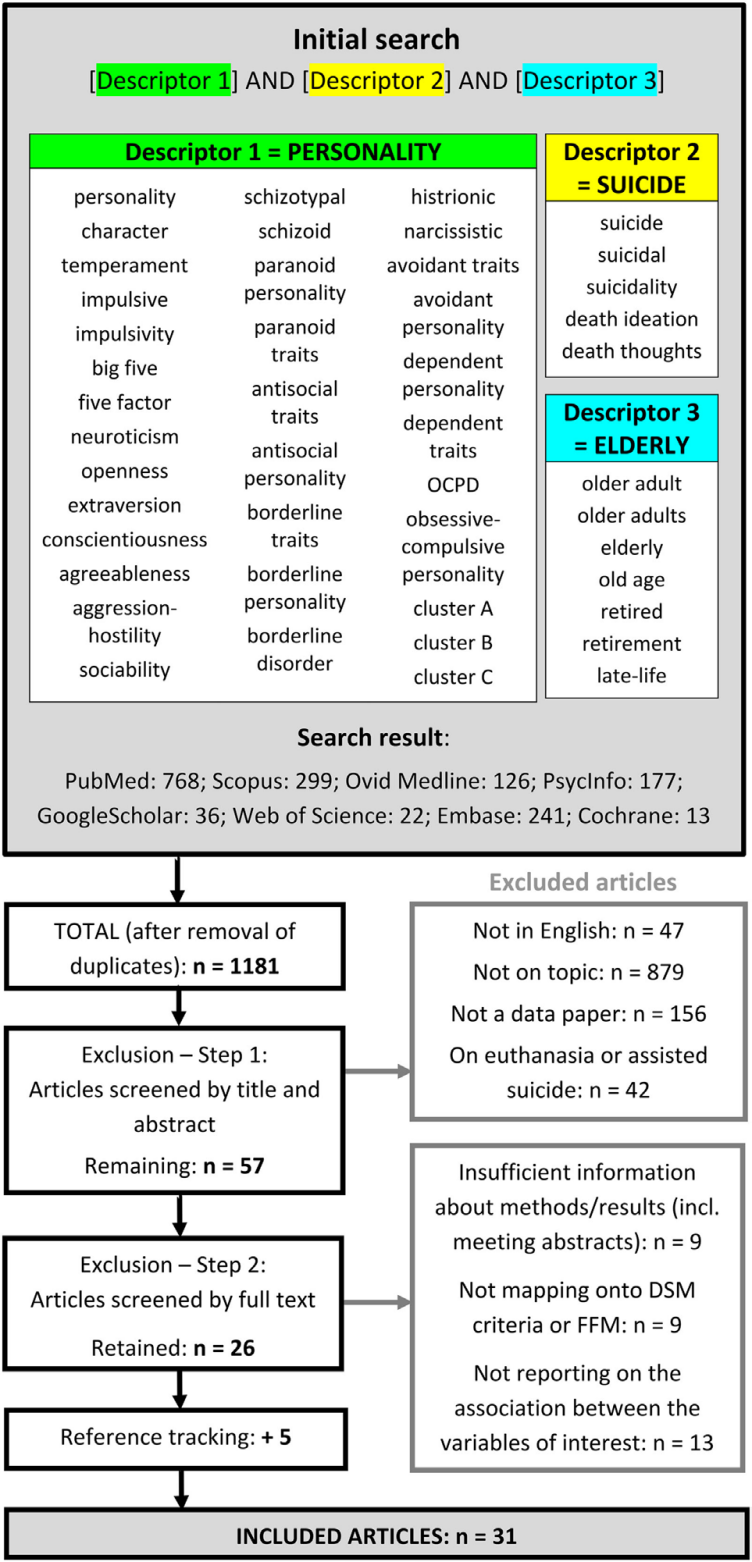
Flow diagram of the studies retrieved for the review. The key words under each descriptor were separated by “OR” for the search. On PubMed, corresponding MeSH terms were added to each descriptor field: Personality, Personality Disorders, Human Characteristics and Impulsive Behavior to Descriptor 1; Suicide to Descriptor 2, and Aged to Descriptor 3. Abbreviation: FFM, Five-Factor Model.

A systematic search was conducted using PubMed, Ovid, Embase, PsycInfo, Cochrane, Web of Science, Scopus, and Google Scholar by cross-referencing three descriptor fields with the following key words (Figure 1): personality (34 key words); suicide (5 key words); and elderly (7 key words). Key words were searched in title and abstract when possible, or else in abstract only (Ovid, PsycInfo), and finally in title only where none of the above were available (Google Scholar, Web of Science). In PubMed, where the added ability to use MeSH terms is available, we complemented our search formula with the following MeSH terms: “Personality/psychology,” “Personality Disorders/psychology,” “Human Characteristics,” and “Impulsive Behavior” were added to the descriptor *personality*, “suicide” was added to the descriptor *suicide* and “aged” to the descriptor *elderly*. In Embase, search terms were both used as free text and mapped on Emtree terms. Reference tracking was done on all included articles to check for additional publications that met inclusion. The search encompassed all articles published through January 31, 2017, with no beginning time limit and no restrictions on publication status. Our methodology and inclusion criteria were defined and documented at the beginning of the project. Publications were included if written in English and describing a research study. In addition, articles had to report an assessment of personality specifically, related to any combination of death by suicide, attempted suicide and suicidal ideation. The suicidal behavior/ideation described could not pertain to assisted suicide or euthanasia. Personality had to be assessed *via*, or map onto the Five-Factor Model (FFM) or DSM/ICD personality constructs. Studies where personality was not reported separately from other psychiatric diagnoses were excluded, as were all studies not distinguishing between older adults and other age groups, or between suicidal behavior and para-suicide. Older age was defined as equal to or above 50 years. Finally, included articles had to contain sufficient data to evaluate the quality of their findings (e.g., this was not the case for meeting abstracts). In addition to these initial criteria, we later refined the definition of research study to those having a minimum *N* of 20 to exclude case reports and small qualitative studies oversampling clinically illustrative cases with weakly generalizable data. The first author, AS, performed the initial screening and then reviewed eligibility of potentially includable articles with AD based on their full text. Of the 1,181 articles found, 1,124 were excluded during the initial screening for one of the following reasons: not written in English (47 articles), not on topic (including all articles about para-suicide/deliberate self-harm; 879 articles), not a data paper (156 articles), having a euthanasia/assisted suicide focus (42 articles). From the 57 remaining articles, further 31 were excluded based on their full text, either because they contained an insufficient amount of information about methods or results (9 articles), were not mapping onto DSM or FFM personality constructs (9 articles), or were not reporting on the association between personality and suicidal behavior/ideation in the elderly (13 articles). Twenty-six articles were retained for the review, and five more were found by reference tracking.

## Results

### Study Characteristics

#### General Characteristics

Of the 31 included articles (Tables 1–4), one was from 1976 (37), and all others were published after 1990, with seven articles between 1990 and 2000 (38–44), 13 articles between 2001 and 2010 (45–57), and 10 articles between 2011 and 2016 (58–67). The association between personality and either death by suicide, attempted suicide or suicidal ideation was the primary outcome in 18 articles (39, 41, 43, 45, 46, 48, 50, 51, 53–56, 59, 61, 63, 65–67), while the other 13 articles reported on personality in relation to a different study focus (37, 38, 40, 42, 44, 47, 49, 52, 57, 58, 60, 62, 64). Except for one qualitative study on death by suicide (56) (displayed in Table 2), all studies were quantitative. Five studies followed a purely descriptive design, without control groups (40, 42, 50, 52, 56), and only nine of the remaining studies included non-suicidal psychiatric controls (39, 43, 44, 46–48, 51, 53, 57). Seventeen articles reported categorical personality disorder diagnoses (Tables 1–3), and 14 measured dimensional personality traits (Tables 4 and 5), while one article utilized both (displayed in both Tables 2 and 4) (59). Dimensional measures were used in an increasing fraction of articles over time: in 8/21 articles between 1976 and 2010 versus 6/10 from 2011 to 2016.

**Table 1:** Studies with categorical measures reporting globally on personality disorders.

	Study	Sample	Age (years)	PD assessment	Summary of results
Death by suicide	Henriksson et al., 1995 (42) Finland Retrospective cohort study, descriptive	229 suicides of which 43 older suicides	≥60	Interviews with relatives and doctor (DSM-III-R)	*Prevalence of PDs in elderly*: suicide victims: 14%; younger suicides: 34%

	Hunt et al., 2006 (52) England and Wales National clinical survey, descriptive	41,959 suicides of which 597 older suicides	≥65	Retrospective diagnosis by consultant psychiatrist (ICD-10)	*Suicides with PD*: 19% (<25 years) 15.3% (25–34 years) 10.8% (35–44 years) 7.8% (45–54 years) 3.3% (55–64 years) 4.0% (65–74 years) 2.3% (≥75 years)

	Qin, 2011 (58) Denmark Population study, retrospective	1,169 suicides (4,175 older) 4,231,219 population controls (193,251 older)	>60	Coded medical records (ICD-10)	*Prevalence of PDs as principal diagnoses*: Suicides: M: 1.5%, F: 4.8% Controls: M: 0.2%, F: 0.5%

Suicide and attempt	Lawrence et al., 2000 (44) Western Australia Hospitalization-based population study, retrospective	447 suicides 1,596 attempted suicides Control values: population distribution	≥60	Medical records (ICD-9)	*PD rate ratios*: Suicide: M: 2.3, F: 12.3 Attempt: M: 2.4, F: 11.8 *Risk ratio (proportional hazards regression of PDs as risk factors)*: Suicide: 2.1 Attempt: 10.2

Attempted suicide	Nieto et al., 1992 (38) Spain, Barcelona Retrospective comparative study	257 suicide attempters with medically serious attempts of which 38 elderly	≥65	Medical records (DSM-III-R)	*PDs with no Axis-I comorbidities in attempters aged ≥65*: 5.2% *All PDs in three attempter age groups*: ≥65 years (*n* = 38): 6 (15.8%) 31–64 years (*n* = 120): 25 (21.8%) 0–30 years (*n* = 99): 45 (45.5%) Oldest group different from youngest group (*p* = 0.003)

	Kunik et al., 1993 (39) USA (PA) Case–control study, cross-sectional	143 patients with major depression of which 37 with PD	≥60	Consensus conference (DSM-III-R)	*History of attempts*: patients with PD: 35% depressed controls: 20% *Correlation (attempts-PD)*: χ^2^(1) = 3.8, *p* = 0.06

	Draper, 1994 (40) Sydney, Australia Retrospective cohort study	69 attempted suicides	≥65	Medical records (DSM-III/III-R, ICD-9)	*Any PD diagnosis*: 26% as secondary diagnosis

	Miret et al., 2010 (57) Madrid, Spain Hospitalization-based population study, cross-sectional	1,970 attempted suicides, from which 113 late-life attempts	≥55	Medical records (ICD-9)	*Prevalence of PDs as principal diagnoses*: Older attempters: *n* = 10 (8.8%) Younger attempters: *n* = 171 (22.6%)

*PD, personality disorder, M, males, F, females*.

**Table 2:** Categorical findings on separate personality disorder diagnoses in death by suicide, and qualitative findings.

	Study	Sample	Age (years)	PD assessment	Summary of results
DSM/ICD	Harwood et al., 2001 (45) England Psychological autopsy study; case–control study in a subsample	100 elderly suicides 54 included in case–control part Controls: 54 age- and gender-matched natural deaths	≥60	Informants (ICD-10)	*Prevalence of PD*: Suicides: 14.8%, controls: 3.7% *Prevalence of PD trait accentuation*: Suicides: 33.3%, controls: 13% *PDs (n *=* 16)*: Anankastic (*n* = 4); dissocial (*n* = 4); histrionic (*n* = 4); other (*n* = 3); mixed (*n* = 5) *PD trait accentuation (n *=* 28)*: Anankastic *n* = 19; anxious *n* = 13; dependent *n* = 9; histrionic *n* = 6; other *n* = 10 *Case–control part*: Correlation with suicide (Fisher’s exact test): Anankastic traits: *p* = 0.012 Anxious traits: *p* = 0.015

	Harwood et al., 2006 (50) England Psychological autopsy study, descriptive	23 suicides without ante-mortem psychiatric diagnoses No controls	≥60	Informants (ICD-10)	*PD (n *=* 1)*: dissocial (*n* = 1) *PD trait accentuation (n *=* 10)*: Anankastic (*n* = 8); paranoid (*n* = 3)

	Ciompi, 1976 (37) Switzerland Longitudinal study in psychiatric patients	1,891 deaths between age 57 and 77 years, of which 266 patients with PD (14%) Control values: average population distribution	≥57	Medical records (psychodynamic nosology of the time)	*Patients with PD who died by suicide*: Of all male deaths = 13 (6.8%) Of all female deaths = 4 (5.4%) *Control values in general population*: Suicides out of male deaths = 1.8% Suicides out of female deaths = 0.7% *Most frequent PDs in suicides*: Psychopathic and neurotic PDs

Qualitative	Kjølseth et al., 2009 (56) Norway Qualitative psychological autopsy study	23 older suicide victims No controls	≥60	Qualitative interviews	*Life history*: hard-working achievers, source of help/advice for relatives *Personality traits*: stubborn, willing to do everything by themselves *Relationships*: introverted, distant or egoistic, with strong self-control

*PD, personality disorder*.

**Table 3:** Categorical findings on suicidal ideation.

	Study	Sample	Age (years)	Assessment	Summary of results
DSM-IV-TR	Segal et al., 2012 (59) USA (CA) Cross-sectional	109 community-dwelling older adults evaluated for SI	≥65	SI scale: GSIS PD scale: Coolidge Axis-II Inventory	*Positive correlation with SI*: all PDs except histrionic *Positive predictor of SI*: borderline PD *Negative predictor of SI*: histrionic PD
	Segal et al., 2015 (66) USA (CA) Cross-sectional	*Positive correlation of SI with former PDs*: depressive PD

	Jahn et al., 2015 (65) USA (CA) Cross-sectional	143 community-dwelling older adults evaluated for SI	≥65	SI scale: GSIS PD scale: SCID-II-PQ	*Positive correlation with SI (in decreasing order)*: avoidant, schizotypal, depressive, borderline, schizoid, dependent, passive-aggressive, and paranoid personality traits *No significant correlation*: obsessive-compulsive, narcissistic, histrionic, and antisocial traits

	Eades et al., 2016 (67) USA (CA) Cross-sectional	102 community-dwelling older adults evaluated for SI	≥61	SI scale: GSIS PD scale: Coolidge Axis-II inventory	*Positive correlation with SI (in decreasing order)*: Depressive, schizoid, schizotypal, passive-aggressive, avoidant, paranoid, borderline, dependent, obsessive–compulsive, and self-defeating *No significant correlation*: histrionic, antisocial, sadistic

	Morse and Lynch, 2004 (47) USA Cross-sectional	63 depressed inpatients, from which 17 with PD	≥64	SI scale: ASIQ PD scale: WISPI	*SI*: no significant correlation with total personality disorders

DSM-III & -IV	Heisel et al., 2007 (53) Canada Cross-sectional, case–control study	538 depressed elderly; 20 with narcissistic PD (*n* = 13)/PD traits (*n* = 7)	≥65	SI scale: HRSD suicide item PD diagnosis: medical records	*Narcissistic PD*: positive predictor of SI *Narcissistic PD traits*: not significant predictors

*PD, personality disorder; SI, suicidal ideation; GSIS, Geriatric Suicide Ideation Scale; SCID-II-PQ, Structured Clinical Interview for DSM-IV-TR Axis II—Personality Questionnaire; ASIQ, Adult Suicide Ideation Questionnaire; WISPI, Wisconsin Personality Disorders Inventory IV; HRSD, Hamilton Rating Scale for Depression*.

**Table 4:** Characteristics of studies with dimensional findings (see Table 5 for a summary of findings).

Study		Sample	Age (years)	Assessment
Suicide	Duberstein et al., 1994 (41) USA (NY, Rochester) Psychological autopsy study	52 suicides 30 healthy controls	≥50	NEO-PI

	De Leo et al., 2013 (60) Australia (QLD, NSW) Case–control study	261 suicides, of which 73 older 182 sudden death controls of which 79 older	≥60	NEO-FFI
	Draper et al., 2014 (63) Australia (QLD, NSW) Case–control study			

Suicide, attempted suicide	Tsoh et al., 2005 (49) China (Hong Kong) Case–control study	67 suicide victims 66 suicide attempters 91 healthy controls	≥65	NEO-FFI

	Useda et al., 2007 (55) USA (NY, Rochester) Case–control study	60 depressed attempters 43 suicides	≥50	NEO-PI-R

Attempted suicide	Seidlitz et al., 2001 (46) USA (NY, Rochester) Case–control study	45 depressed attempters 36 depressed controls	≥50	NEO-PI-R (emotion facets)

	Wiktorsson et al., 2013 (61) Sweden Case–control study	72 hospitalized attempters 288 healthy controls	≥75	EPI (N and E subscales)

	Chan et al., 2014 (62) China (Hong Kong) Case–control study	77 suicide attempters 99 healthy controls	≥65	NEO-FFI NEO-PI facets

Attempted suicide, suicidal ideation	Duberstein et al., 2000 (43) USA (NY, Rochester) Case–control study	45 depressed attempters 32 patients with recent suicidal ideation (1 week) 61 patients without recent SI 36 depressed controls	≥50	SSI NEO-PI-R

	Useda et al., 2004 (48) USA (NY, Rochester) Case–control study	67 depressed attempters 43 depressed controls + Assessment of severity of SI	≥50	SSI NEO-PI-R

Suicidal ideation	Heisel et al., 2006 (51) USA (NY, Rochester) Case–control study	32 depressed suicide ideator 101 depressed controls	≥50	SSI NEO-PI-R

	Hirsch et al., 2007 (54) USA (NY, Rochester) Case–control study	462 older adults recruited from a primary care setting of which 37 suicide ideators	≥65	SCID and HRSD (SI questions) NEO-FFI

	Segal et al., 2012 (59) USA (CO, Colorado Springs) Cross-sectional	109 community-dwelling elderly	≥65	GSIS NEO-FFI

	Kim and Ahn, 2014 (64) USA (LA) Cross-sectional	220 community-dwelling elderly Korean immigrants	≥60	SSI EPQR (N subscale)

*SI, suicidal ideation; NEO-PI, NEO Personality Inventory, NEO-FFI, NEO Five-Factor Inventory; EPI, Eysenck Personality Inventory; EPQR, Eysenck Personality Questionnaire; SSI, Scale for Suicide Ideation; SCID, Structured Clinical Interview; HRSD, Hamilton Rating Scale for Depression; NEO-PI, NEO Personality Inventory; NEO-PI-R, NEO Personality Inventory Revised; GSIS, Geriatric Suicide Ideation Scale*.

**Table 5:** Summary of dimensional findings on death by suicide, attempted suicide, and suicidal ideation.

Domains; facets	Suicide victims	Suicide attempters	Suicide ideators
**Neuroticism**	> HC (41, 60), ≈ HC (49)^a^^,^^b^ < SA (55)^a^^,^^b^, ≈ SA (49)^a^ < ySV (60) (63)^c^	> HC (62), ≈ HC (49)^a^^,^^b^ (61)^b^ ≈ DC (43)	> HC (59) (64)^a^ > DC (43), ≈ DC (51)^a^^,^^b^
*Depression*	> *HC* (41); < *SA* (55)^*a*^^*,*^^*b*^	≈ *DC* (46, 48)	> *DC* (48)
*Self-consciousness*	> *HC* (41); < *SA* (55)^*a*^^*,*^^*b*^	> *DC* (48), ≈ *DC* (46)	≈ *DC* (48)
*Impulsiveness*	≈ *HC* (41); < *SA* (55)^*a*^^*,*^^*b*^	> *HC* (62); > *DC* (48)	≈ *DC* (48)
*Vulnerability*	≈ *HC* (41); < *SA* (55)^*a*^^*,*^^*b*^	> *DC* (48)	≈ *DC* (48)
*Anxiety/negative affect^g^*	≈ *HC* (41); < *SA* (55)^*a*^^*,*^^*b*^	< *DC* (46), ≈ *DC* (48)	≈ *HC* (54)^*a*^^*,*^^*b*^^*,*^^*g*^; ≈ *DC* (48)
*Anger–hostility*	≈ *HC* (41); ≈ *SA* (55)^*a*^^*,*^^*b*^	> *HC* (62)^*e*^, ≈ *HC* (62)*^f^* ≈ *DC* (46, 48) *More attempts* (46) *Lower lethality and intent* (46)	≈ *DC* (48)

**Extraversion**	< HC (60), ≈ HC (41) (49)^a^^,^^b^ ≈ SA (49)^a^ (55)^a^^,^^b^ ≈ ySV (60, 63)	< HC (62)^e^, ≈ HC (49)^a^^,^^b^ (61)^b^ (62)^f^ < DC (43) Fewer attempts (43)	≈ HC (59) ≈ DC (51)^a^^,^^b^
*Positive emotions/positive affect^g^*	*No data*	< *DC* (46) (48)^*a*^^*,*^^*b*^ *Fewer attempts* (46) (48)^*a*^^*,*^^*b*^	< *HC* (54)^*a*^^*,*^^*b*^^*,*^^*g*^; < *DC* (48)
*Warmth*	*No data*	≈ *DC* (46, 48)	< *DC* (48)^*a*^^*,*^^*b*^
*Gregariousness/sociability*^^g^^	*No data*	≈ *DC* (48)	≈ *HC* (54)^*a*^^*,*^^*b*^^*,*^^*g*^; ≈ *DC* (48)

**Openness to experience**	< HC (41), ≈ HC (49)^a^^,^^b^ (60) ≈ SA (49)^a^ (55)^a^^,^^b^ > ySV (41) (60) (63)^c^	≈ HC (49)^a^^,^^b^ (62) ≈ DC (48)	≈ HC (59) > DC (43) (51)^a^^,^^b^, ≈ DC (48)
*Openness to esthetics*	< *HC* (41)	≈ *DC* (48)	*No data*
*Openness to action*	< *HC* (41); < *ySV* (41)	≈ *DC* (48)	*No data*

**Conscientiousness**	≈ HC (41) (49)^a^^,^^b^ (60) > SA (49)^a^ (55)^a^^,^^b^ > ySV (60) (63)^d^	< HC (49)*a* (62)^e^, ≈ HC (62)^f^ ≈ DC (43)	≈ HC (59) ≈ DC (48) (51)^a^^,^^b^
*Dutifulness*	> *SA* (55)^*a*^^*,*^^*b*^	≈ *DC* (48)	*No data*
*Achievement-striving*	> *SA* (55)^*a*^^*,*^^*b*^	**≈** *DC* (48)	*No data*
*Self-discipline*	> *SA* (55)^*a*^^*,*^^*b*^	**≈** *DC* (48)	*No data*
*Deliberation*	> *SA* (55)^*a*^^*,*^^*b*^	**≈** *DC* (48)	*No data*

**Agreeableness**	≈ HC (41) (49)^a^^,^^b^ (60) ≈ SA (49)^a^ (55)^a^^,^^b^ ≈ ySV (60 63)	≈ *HC* (49)^a^^,^^b^ (62) ≈ *DC* (43)	≈ HC (59) < DC (43), ≈ DC (48) (51)^a^^,^^b^
*Modesty*	*No data*	> *DC* (48)^*a*^^*,*^^*b*^ *More attempts* (48)^*c*^	> *DC* (48)^*a*^^*,*^^*b*^

*All five domains of the Five-Factor Model and their facets with significant findings are displayed with corresponding references*.

*>, higher levels than; <, lower levels than; ≈, not statistically different from; HC, healthy controls; DC, depressed controls; SA, elderly suicide attempters; ySV, younger suicide victims*.

*^a^Result after adjusting for general health status (physical and/or cognitive)*.

*^b^Result after adjusting for psychiatric status (any Axis-I pathology or depression)*.

*^c^In psychiatric subgroup*.

*^d^In non-psychiatric subgroup*.

*^e^Males*.

*^f^Females*.

*^g^Subcomponents derived from the NEO-FFI*.

#### Age Cutoffs

Age cutoffs ranged from 50 to 75 years, with a majority of studies including either participants aged 60 years (11 articles) or 65 years (12 articles) and older. Only one study had an age cutoff as high as 75 years (61). Mean age was, however, above 70 years in 17/24 articles where these data were available (several studies indicated the percentage of elderly subjects in different age subgroups without reporting a global value for mean age).

#### Personality Measures

Even though 17 articles reported on categorical personality disorders, findings about specific personality diagnoses remained scarce in death by suicide and attempted suicide: 6/17 studies reported only global data on personality disorders (38–40, 44, 52, 57), and two others solely specified BPD in addition to total personality disorders (42, 58) (Table 1). Of the remaining nine publications, findings on death by suicide were reported in three articles (37, 45, 50), two of which were based on the same study (Table 2). No article assessed distinct personality disorders in attempted suicide. Suicidal ideation was assessed by six studies (47, 53, 59, 65–67), of which four were based on the same population (community-dwelling older adults in Colorado, USA) (Table 3). One study on suicidal ideation focused exclusively on narcissistic personality disorder (53).

Of the 14 articles measuring dimensional personality traits (Table 4), two based their findings on the Eysenck Personality Model, assessing only Neuroticism in one case (64), and Neuroticism and Extroversion in the other (61). Based on the reasonable levels of correlation established by Costa and McCrae between Eysenck’s Neuroticism and Extroversion and the Five-Factor neuroticism and extraversion domains (68), we considered these constructs equivalent when reporting results. All other studies used the FFM. Most of them exclusively included results for the five main factors, however, 6/14 articles reported findings on the facet level. Subcomponents derived from the NEO-FFI (NEO Five Factor Inventory) such as *negative emotions*, were interpreted together with the most highly correlated facets derived from the NEO-PI-R (NEO Personality Inventory Revised), in this case, *anxiety* (69). Three studies with facet-level analyses focused only on specific sets of facets (46, 54, 62). Overall, five articles provided dimensional data on death by suicide (41, 49, 55, 60, 63), seven on attempted suicide (43, 46, 48, 49, 55, 61, 62), and six others on suicidal ideation (43, 48, 51, 54, 59, 64). Four articles reported on multiple suicidal outcomes (43, 48, 49, 55). Two articles reported findings based on the same study (60, 63). Seven out of the 14 studies based on dimensional models had overlapping samples (elderly patients recruited in the teaching hospitals of the University of Rochester, NY, USA), and two others shared a recruitment source (community-dwelling older adults in Colorado, USA) (Table 4). The dimensional findings, however, came from at least three different sample sources for each suicidal outcome (death, attempt, and ideation).

#### Definition of Death by Suicide, Attempted Suicide, and Suicidal Ideation

Not surprisingly, older suicide victims were the most uniformly defined study group, with the obvious inclusion criterion being death by suicide occurring in old age. One study comparing individuals who died by suicide to older attempters also excluded suicide victims with a lifetime history of attempt (55).

While most articles did not define suicide attempt, two studies reportedly used the definition of the World Health Organization (57, 61): “Those situations in which a person has performed a life-threatening act with the intent of putting his or her life into danger or giving the appearance of such an intent” (70). Other studies defined suicide attempts as any intentional self-destructive act, without necessitating an expressed intent to die, blurring the line between suicide and para-suicide (40, 46, 48). Most studies used the presence of lifetime attempt history to define their attempters, whereas others necessitated a recent attempt, only including older individuals from either inpatient or emergency units (38, 44, 49, 57, 61, 62). In 4/6 of these cases, history of prior attempts was either controlled for statistically (49, 57) or excluded from the control group (61, 62).

Some studies included passive death wish in their definition of suicidal ideation (43, 51). Further adding to the samples’ heterogeneity, studies of elderly suicide ideators neither excluded nor controlled for subjects with past history of suicide attempts. Thus, findings about suicide ideators should be interpreted with caution, since they reflect more a global suicidal risk than pure suicidal contemplation.

### Correlates and Possible Mediators

#### Gender

An established pattern in the literature is that men are more likely to die by suicide while women are more likely to have non-fatal attempts and subsequent mental health contact (71). Thus, a selection bias cannot be excluded in the 14 studies whose suicidal sample was exclusively recruited based on previous contact with psychiatric health services (37, 39, 40, 43, 44, 46–49, 51–53, 55, 57, 61, 62) as these studies might have oversampled women. While personality pathology increased with late-life suicidal behavior in both genders in most studies, several reported a higher female to male ratio. Lawrence and colleagues found a fivefold higher rate ratio in females with personality pathology than in males for both attempted suicide and death by suicide (44). Qin, whose population study assessed psychiatric illness *via* the last hospitalization-based principal diagnosis, also reported a threefold higher prevalence of personality pathology in female versus male suicides (58). Not supporting these findings, one case–control study, with separate regression models for elderly male and female attempters, reported that neuroticism was a significant, positive predictor in men but not in women (62). In addition, the Lausanne Study indicated a 1.3 male to female ratio for the prevalence of suicide in former inpatients with personality disorders (37), and Harwood and colleagues’ psychological autopsy study did not find a significant gender difference in personality disorder rates (45). The majority of case–control studies included in the review controlled for the potential effect of gender, with only four that did not, or for which this information was missing (40, 47, 65, 66).

#### Depressive Disorders

Depressive disorders in old age have been separately associated with both suicidal behavior (28, 72), and personality disorders (9, 73). Personality disorders co-occur in 10–30% of depressed elderly, with Cluster C disorders being the most frequent and Cluster B disorders the rarest (9). Patients with personality disorders were found to be up to four times more likely to suffer from the persistence or the re-emergence of depressive symptoms (39, 47) and were younger at first onset (39). Morse and Lynch found that the presence and not the severity of personality pathology was correlated with poor prognosis of depressive symptoms in the elderly (47). Of the studies included in this review, only 4/12 studies of death by suicide (49, 55, 58, 60) and 7/12 of suicidal ideation (43, 47, 48, 51, 53, 54, 65) controlled for depression when evaluating the relationship between personality and suicide risk. Studies of attempted suicide were more diligent in this regard, their recruitment source most often being pools of depressed patients. Only one comparative study on attempted suicide lacked any form of adjustment for depressive disorders (44). In 143 older primary care patients, Jahn and colleagues found that depressive symptoms mediated the effect of pathological personality traits on suicidal ideation (65). While none of the studies investigated whether depressive symptoms mediate the effect of personality disorders on suicidal behavior, Nieto and colleagues found that personality disorders occurred independently of all Axis-I diagnoses in 5.2% of older adults aged 65 or above who made a medically serious suicide attempt (*N* = 38) (38).

#### Interpersonal Functioning

Impaired interpersonal functioning is the core feature of personality disorders and a risk factor for suicidal behavior in late-life (74). It is therefore expected to explain the effect of personality pathology on suicidal behavior and ideation. Kunik and colleagues found that depressed inpatients were more likely to be single, separated or divorced if they were diagnosed with a comorbid Axis-II disorder (39). In light of the finding that older adults most often tend to give warning signs about their suicidal intent to their next-of-kin (75), it may be particularly difficult for socially isolated elderly to reach out for help when needed. Conceptualizing the question from the perspective of the interpersonal theory of suicide, Jahn and colleagues identified perceived burdensomeness and thwarted belongingness as mediators between personality disorders and suicidal ideation in older adults aged 65 years or over (*N* = 143) (65). However, Eades found a direct relationship of pathological personality traits and suicidal ideation that was stronger than through either thwarted belongingness or perceived burdensomeness in a community sample of 102 individuals aged 60 or above (67).

#### Environmental Stressors

The longitudinal findings of the Lausanne Study suggest higher late-life suicide rates in psychiatric patients who displayed a “specific vulnerability to environmental stress, evident already years ago during their first admission” (37). Harwood and colleagues investigated personality traits in a subsample of individuals who died by suicide and did not have a psychiatric diagnosis (*N* = 23). Their findings indicated that personality trait accentuation was present in 44% of suicide victims, often concomitantly with recent life events that were thought to have triggered the suicidal act (50). Consistent with Mann’s stress–diathesis model of suicide, these findings support the presence of an increased chronic vulnerability that is exacerbated by negative life events in older individuals with already subclinical levels of personality pathology (7).

### Synthesized Findings

#### Prevalence of Any Personality Disorder in Late-Life Suicide

A psychological autopsy by Harwood and colleagues and a retrospective cohort study by Henriksson and colleagues estimated that respectively, 14 and 16%, of adults who died by suicide after the age of 60 had personality disorders (42, 45). These rates are somewhat higher than the 10% community prevalence of personality disorders in adults above 50 years reported by a meta-analysis (76), but the difference remains small considering possible confounding with other psychopathology. Including only principal diagnoses in their analysis, Hunt and colleagues’ and Qin’s population studies found a much lower prevalence of personality disorders in late-life suicide victims, namely between 1.5 and 5% (52, 58). However, these latter findings are likely less accurate since personality disorders are rarely considered principal diagnoses in this age group (42). Examining personality trait accentuation in addition to personality disorders, Harwood and colleagues’ psychological autopsy study found that subthreshold accentuated personality traits were present in 28% of older adults who died by suicide (*N* = 100) (45). Thus, altogether, 44% of the suicide victims in Harwood and colleagues’ sample had some level of personality pathology.

Despite these relatively high rates of pathological personality disorders and traits in late-life suicide, all studies with younger comparison groups found decreasing prevalence of personality pathology with age (42, 52, 57, 58). The prevalence of any personality disorder was reported to be 2.4 and 2.6 times lower, respectively, in elderly suicide victims aged 60 or above (42), and suicide attempters aged 55 or above (57) than in the younger comparison groups. Both Hunt and colleagues and Harwood and colleagues found that the prevalence of personality disorders further decreased between 60 and 75 years of age in elderly suicide victims (respectively, from 4.0 to 2.3% for personality disorders as primary diagnoses and from 19.7 to 10.6% at any detectable level) (45, 52).

This significant decrease in older people may be due to the attenuation of symptoms of certain commonly diagnosed personality disorders such as BPD, and/or to a survival bias related to increased premature mortality due to personality pathology. Furthermore, a detection bias is likely in old age given that personality disorders may be masked by overlapping cognitive deficits in a subset of older patients seeking psychiatric care (77). In turn, suicidal behavior may also be under-detected in old age: studies highlighted a decreased number of psychiatric hospitalizations of older adults, both before dying by suicide (58), and after a suicide attempt (44). This may be in part related to diagnostic biases. Indeed, comorbid cognitive deficits, delirium or other organic conditions often lead to medical rather than psychiatric hospitalizations in the elderly with decompensated psychopathology (78); primary health care providers have been found less likely to assess psychopathology in older adults than in younger patients (79); suicidal and death ideation are perceived as part of normal aging by many clinicians (80); and social isolation can cause low-lethality suicide attempts to go unnoticed.

#### Association of Personality Disorder Diagnosis With Suicidal Behavior and Ideation in the Elderly

##### Death by Suicide

A positive correlation between personality pathology and death by suicide was supported by most studies, although none were designed to separate unique contributions of personality disorders from those of other types of psychopathology. Harwood and colleagues found both personality disorders and traits being more frequent in a subsample of 54 elderly suicide cases compared to age- and sex-matched controls deceased from natural causes (45). Qin determined a respectively sixfold and ninefold higher prevalence of personality disorders in older females and males who died by suicide compared to general population controls (58). In a sample of psychiatric patients aged 60 years or older, Lawrence and colleagues found that personality disorders had the fifth highest suicide rate among all principal psychiatric diagnoses in the elderly, which was more than twofold higher than in the age-matched general population of Western Australia (risk ratio of 2.1, *n* = 447) (44). A single longitudinal study examined the prevalence of suicide in individuals with severe personality disorders: the Lausanne Study was conducted in Switzerland from 1963 to investigate aging in all former psychiatric inpatients of the Lausanne University Psychiatric Hospital (*N* = 5,661). The investigator found that personality disorders were the third most common baseline psychopathology after mood disorders and alcohol dependence among the 107 suicides that were carried out by patients between 57 and 77 years of age (37). The prevalence of suicide in the subgroup of patients with personality disorders was 6.8% in males and 5.4% in females (*n* = 266), respectively, more than threefold and fivefold higher than in the age- and gender-matched general population. However, the severe pathology that triggered hospitalizations in this group of patients precludes generalization of these findings to personality disorders observed in the community.

##### Attempted Suicide

Examining only primary diagnoses, Miret and colleagues reported a prevalence of 8.8% for personality disorders in hospitalized patients aged 50 and older who had made a recent suicide attempt (*N* = 113) (57). Draper found a higher prevalence (26%) considering secondary diagnoses mentioned in the hospital records of 69 attempters aged 65 or above (40). In addition, Nieto and colleagues reported that personality disorders were present without comorbid Axis-I disorders in 5.2% of 38 older adults aged 65 or above who made a medically serious suicide attempt (38). This fraction was lower than in young attempters (30 or younger) from the same study (45.5%, *p* = 0.003), but did not differ significantly from middle-aged attempters (31–65 years) (21.8%). Lawrence and colleagues reported a risk ratio of 10.2 for suicide attempts given a principal diagnosis of personality disorder (*n* = 1,596), almost five times higher than the 2.1 risk ratio for death by suicide (44). Examining psychiatric patients with personality disorders (*n* = 37), Kunik and colleagues found a 35% lifetime prevalence of attempted suicide. This prevalence, however, was not significantly different from the 20% seen in the clinical control group without personality disorders [*n* = 117; χ^2^(1) = 3.76, *p* = 0.06] (39).

##### Suicidal Ideation

Studies found a positive association with suicidal ideation and almost all categories of pathological personality traits in late life, with the exception of histrionic and sadistic traits (47, 59, 65–67). None of these studies, however, excluded suicide ideators with a past history of attempt. No correlation was found between the severity of pathological personality traits and the severity of suicidal ideation (47).

#### ICD/DSM Personality Disorders in Late-Life Suicide

Using ICD-8 personality diagnoses the abovementioned Lausanne Study found that neurotic and psychopathic (antisocial) personality disorders were the most prevalent in former psychiatric patients who died by suicide after the age of 57 (37). In the psychodynamic nosology of the time, a “neurotic personality organization” included hysterical (i.e., histrionic), depressive-masochistic (best mapping onto self-defeating personality from DSM-III), obsessive (mapping onto obsessive–compulsive), as well as avoidant personalities (81). These findings support a role of Clusters B and C in late-life suicide, which is consistent with the more recent results detailed below. As mentioned, no study reported data on specific personality disorders and attempted suicide in the elderly.

##### Cluster A

###### Paranoid Personality Disorder

In Harwood and colleagues’ secondary analysis of cases without an ante-mortem psychiatric diagnosis (*N* = 23), paranoid traits were the second most frequent (13%) (50). However, in the absence of a control group, this finding remains difficult to interpret given the relatively high prevalence of paranoid personality in the general population (2–5%) (82, 83).

There was nevertheless a positive correlation of suicidal ideation with paranoid personality in all studies that investigated it (47, 59, 65, 67).

###### Schizoid Personality Disorder

There is no evidence linking schizoid personality disorder to late-life suicide, in spite of the documented worsening of schizoid personality with aging (84). To the best of our knowledge, this disorder is not associated with suicidal behavior earlier in life either.

A positive correlation was however found in all studies investigating schizoid traits in suicidal ideation (47, 59, 65).

###### Schizotypal Personality Disorder

No evidence linked schizotypal personality disorder to late-life suicide.

Three studies, with possibly overlapping community samples recruited in Colorado, found a positive correlation with suicidal ideation (59, 65, 67); however, such a relationship was not confirmed by Morse and Lynch in a depressed sample with a different recruitment source (47).

Despite the high co-occurrence of schizotypal personality disorder with BPD in the general population (85, 86), its role in suicidal behavior remains also unclear earlier in life.

##### Cluster B

###### Antisocial (and Dissocial) Personality Disorder

Studies in younger adults have linked antisocial personality to suicide attempts (17, 87, 88) and death by suicide (18). Moreover, antisocial personality traits appear to persist, or only partially remit with increasing age in most individuals (89), suggesting a possible high suicide risk throughout the lifetime, yet data so far remain inconclusive. The Lausanne Study found a high prevalence of psychopathic (antisocial) personality disorder in former patients who died by suicide after the age of 57 (37). Harwood and colleagues found dissocial personality disorder to be among the three most prevalent personality disorders in their study, with 4% of suicide victims reaching full diagnostic criteria for it (45). However, in the case–control part of their study (*n* = 54), the prevalence of antisocial personality did not differ between suicide victims and controls.

While one study (59) found an association between antisocial traits and suicidal ideation in old age, three others did not (47, 65, 67).

###### Borderline (and Emotionally Labile) Personality Disorder (BPD)

In spite of its important role in suicides earlier in life, BPD showed no significant association with death by suicide in old age. Moreover, two studies found a significantly lower prevalence of BPD in both elderly suicides and controls than in younger age groups (42, 58), which is unsurprising given that most features of BPD tend to remit with age (90).

However, all studies investigating suicidal ideation found a positive correlation with borderline personality traits (47, 59, 65, 67). Segal and colleagues also identified borderline traits as the only positive predictor of suicidal ideation in a community sample of 109 adults aged 60 or above (59).

As mentioned earlier, BPD in old age may be subject to a survival bias. In a 27-year long longitudinal study in BPD patients (*N* = 165), Paris and Zweig-Frank reported death by suicide in as many as 10.3% of their sample with a mean age at death of 37.2 years (91). Despite persisting problems with interpersonal functioning, the surviving cohort showed significant improvement, with only five patients meeting full criteria for BPD by the end of the follow-up. Although an increase in attempts’ lethality with advancing age has been reported by Soloff and colleagues (87), Pompili and colleagues’ meta-analysis of suicide in BPD found a higher incidence of suicides in short-term follow-ups, suggesting lower suicidal rates during the chronic phases of BPD (92). Overall, these findings suggest that suicide risk decays, albeit incompletely, in the borderline patients who survive to old age.

###### Histrionic Personality Disorder

Even though histrionic personality is considered to be only mildly impairing and not linked to major emotional disability in the general population (82), Harwood and colleagues found full diagnostic criteria for histrionic personality disorder in 4 out of 100 individuals who died by suicide. Moreover, they found subthreshold histrionic traits in six others, making this personality construct the third most frequent in their sample, and the most prevalent Cluster B disorder (45). As discussed earlier, hysterical (former version of histrionic) disorder was also associated with suicides in the Lausanne Study (37).

These findings contrast with the absence of positive correlation found between suicidal ideation in old age and histrionic personality (47, 59, 65, 67). This disorder was in fact the only negative predictor of suicidal ideation in Segal and colleagues’ study (59), corroborating a potential protective impact on suicide risk.

In younger adults, a protective effect of comorbid histrionic personality disorder was found in an adult sample of female attempters with BPD (93).

###### Narcissistic Personality Disorder

To date, no definite association has been found between narcissistic personality disorder and death by suicide in old age. However, in a qualitative psychological autopsy study conducted by Kjølseth and colleagues with the informants of 23 elderly who died between 65 and 90 years of age in Norway, the majority of suicide victims were described as self-centered over-achievers, with a need to control others, sometimes despite generating conflicts due to their authoritarianism (56).

Narcissistic personality disorder was positively correlated with late-life suicidal ideation in all studies (47, 59, 67) but one (65). In addition, it was a predictor of suicidal ideation in a retrospective database analysis specifically focusing on narcissistic personality in adults aged 65 years and older (*N* = 538) (53).

Narcissistic personality disorder has been identified as a risk factor for suicide in middle-aged adults by a 10-year long longitudinal study reporting a positive correlation between narcissistic personality and number of suicide attempts in a clinical sample of 431 middle-aged adults (aged 18–45 years upon enrollment) (15). In the clinical literature, Kernberg also implicated pathological narcissism in existential crises in the second half of life (94). He discussed how suicide may appeal to narcissistic patients as a means of relieving guilt over past mistakes or missed opportunities for greatness, as “external reality gradually demonstrates [that their grandiose] fantasies are no longer viable.” This risk may likely persist after age 50. However, the low general prevalence of narcissistic personality disorder, estimated at 0.8% (83), suggests that higher-powered studies are needed to detect a potential association.

##### Cluster C

###### Avoidant (and Anxious) Personality Disorder

Despite an absence of subjects meeting full criteria for anxious personality disorder, Harwood and colleagues found a higher rate of anxious personality trait accentuation in suicide victims than in natural death controls, with 13% of suicide victims displaying anxious traits (45).

Furthermore, avoidant personality disorder was positively correlated with suicidal ideation in all studies (47, 59, 65, 67).

Age-related social isolation is possibly more pronounced in avoidant individuals, as the traits related to social ineptitude were found among the disorder’s most stable diagnostic criteria (95). Avoidant personality disorder in the elderly is also highly prevalent in dysthymic disorder (73), an independent risk factor for suicide (96). Some evidence alternatively supports a lifelong risk between avoidant personality disorder and both suicide attempts (97) and death by suicide earlier in life (18).

###### Dependent Personality Disorder

Dependent personality disorder has a high prevalence in the general elderly population (76) and has been associated with suicides and suicide attempts earlier in life (18, 85). Nevertheless, this disorder showed no significant association with death by suicide in old age.

A positive correlation between dependent personality traits and suicidal ideation was found in most (59, 65, 67), but not all studies (47).

###### Obsessive–Compulsive (and Anankastic) Personality Disorder (OCPD)

OCPD displays the strongest association with death by suicide in the existing studies, although the relationship is thin and the studies lack adequate controls. In Harwood and colleagues’ psychological autopsy study (*N* = 100), out of the 44 suicide victims with personality pathology there were 23 cases (52.3%) with anankastic (obsessive–compulsive) personality disorder (*n* = 4) or trait accentuation (*n* = 19). OCPD was also associated with suicide in the case–control part of the study (*n* = 54). In a secondary analysis examining subjects without a psychiatric diagnosis, anankastic personality trait accentuation was present in 8/23 subjects (34.8%) (50). Some of the personality characteristics described by Kjølseth and colleagues’ qualitative findings also map onto OCPD, such as reluctance to accept help from others, self-discipline, high professional competence, hard-work, introversion, and stubbornness (*N* = 23) (56).

Evidence of the association between OCPD and suicidal ideation remains inconsistent, with two studies reporting a positive correlation (59, 67) and two others reporting none (47, 65).

OCPD is the most common personality disorder in the general population, with a prevalence of 3–8% (98). It is also the personality disorder with the highest prevalence (17.1%) among elderly patients with dysthymic disorder (73, 99), which, as mentioned previously, is independently associated with late-life suicide (96). Along with schizoid personality disorder, OCPD is one of only two Axis-II conditions found to become accentuated in old age (84, 100), even in individuals who seemed unimpaired earlier in life (101, 102). Consistent with the qualitative findings of Kjølseth, three OCPD traits related to cognition and interpersonal functioning seem to display a stable and long-lasting pattern: preoccupation with details, rigidity and stubbornness, and reluctance to delegate (95, 103). These characteristics could cause a perception of loss of control in old age stemming from physical and cognitive decline, leaving suicide as a way to regain control. Supporting this hypothesis, two qualitative psychological autopsy reports examined elderly suicide victims who had, respectively, the conviction of having cancer (*n* = 8) or a chronic dyspnea diagnosis (*n* = 14), and both described a similar, rigid personality style in a majority of cases (5/8 and 12/14, respectively) (104, 105). In studies of decision-making, an extreme willingness to wait for delayed rewards has been found in both OCPD patients and high-lethality older attempters, indicating their common tendency to focus on long-term rewards, without considering alternate solutions (106, 107). The extent to which OCPD patients premeditate attempts, however, remains subject to controversy. Whereas some case–control studies in clinical adult samples support the notion of single attempts in OCPD (108), others report a more borderline-like pattern with impulsive suicidal behavior, multiple attempts, and lower intent to die (16). In a series of case reports on high-lethality first time suicide attempters with a double-diagnosis of OCPD and mood disorders, 6/7 cases were aged 56 or above, and all of them described transient, sudden loss of control preceding their suicidal act (109). However, this behavior may be attributed as much to impulsivity as to a general lack of perspective of alternative options.

#### Five-Factor Model

See Table 5 for a summary of findings.

##### Neuroticism

Although high neuroticism has been linked to death by suicide in younger age groups (41), older psychiatric patients who died by suicide displayed lower levels of neuroticism than both their younger counterparts (60, 63), and those who survived their attempt (55). Interestingly, older victims were more similar to healthy controls than they were to attempters. Even though two studies found higher levels of neuroticism in older suicide victims than in healthy controls (41, 60), on the facet level, only trait depression (the tendency to feel sad) and trait self-consciousness (the tendency to be easily intimidated) were higher (41). Suicide victims and healthy controls did not significantly differ in the facets that capture negative emotional reactivity, namely vulnerability (the tendency to panic easily), trait anxiety (the tendency to worry), impulsiveness (the tendency to act impulsively), and anger–hostility (the tendency to get angry easily) (41). Anger–hostility was also the only similar facet between suicide victims and attempters (55).

Among older suicide attempters, two studies conducted in Hong Kong, China, found increased levels of neuroticism compared to healthy controls (49, 62), but this finding did not survive controlling for current major depression, past suicide attempts, physical comorbidities, demographic factors, and life events in one of them (49). Other studies observed no difference from healthy controls (61), or depressed controls (48). Again, differences emerged at the facet level, with higher levels of all facets in attempters than in depressed controls, except for trait anxiety and anger–hostility (48). Lower trait anxiety was in fact found to be a predictor of the attempter status, while lower anger–hostility was associated with fewer attempts, higher lethality of method, and higher intent to die (46). Compared to healthy controls, higher levels of anger–hostility predicted attempter status in men, but not in women, while higher impulsivity was observed in elderly attempters of both sexes (62).

Higher levels of neuroticism unequivocally differentiated elderly suicide ideators from healthy controls (59, 64). However, in depressed samples, only one (43) out of three studies found higher levels of neuroticism in ideators than in controls, with the others reporting no difference (48, 51). Higher trait depression was the only facet that correlated with the severity of suicidal ideation from the neuroticism domain in a sample of depressed elderly (48), indicating that differences may be limited to only isolated facets.

##### Extraversion

Lower levels of extraversion were found in older adults who died by suicide compared to healthy controls in some (49, 60), but not all studies (41). Furthermore, in Tsoh and colleagues’ study, the effect disappeared in the multivariate analysis that controlled for current major depression, past suicide attempts, physical comorbidities, demographic factors, and life events (49). Extraversion did not distinguish between older adults who died by suicide and those who only attempted it (49, 55).

Findings examining elderly suicide attempters and ideators were also inconclusive. Three studies using the same recruitment source found that elderly depressed inpatients who made an attempt at age 50 or older had lower levels of extraversion than depressed controls, both globally, and for the specific facets of gregariousness (seeking company of others), warmth (making friends easily), and positive emotions (tendency to be joyful) (43, 46, 48). In addition, lower positive emotions were identified as an independent predictor of a greater number of attempts (46). However, older suicide attempters did not differ from general population controls on extraversion in two other studies controlling for age, sex, and major depression (49, 61). These inconsistent findings may be explained by sex differences, since lower levels of extraversion were present in elderly male, but not female attempters in a sex-stratified study (62). A sampling bias cannot be excluded, however. Alternatively, given the higher age cutoff in the three latter studies (65–75 years), this inconsistency might arise from a survival bias, as higher levels of extraversion have been associated with a decreased risk of death in the elderly (110).

Similarly to elderly attempters, suicide ideators aged 50 or older differed from depressed controls by reporting lower levels of both warmth and positive emotions in two studies having the same recruitment source as the three studies mentioned earlier (48, 51). They did not differ from healthy controls on extraversion in a community-based sample aged 60 or older (*N* = 109) (59). From the three subcomponents of extraversion derived from NEO-FFI self-reports, positive affect (being joyful), but not sociability (being outgoing and sociable) or activity (tendency to be energetic and busy), was identified as a negative predictor for suicidal ideation (54).

The fact that distinctively socially oriented facets of extraversion (namely, warmth, positive emotions, and gregariousness) differentiated elderly suicide attempters from depressed controls may suggest that a tendency toward social isolation could predispose to suicidal behavior in old age. Even though younger individuals who died by suicide were also found to have lower levels of extraversion than healthy controls (20, 41), in their case, lower levels of activity (being frequently busy) and assertiveness (tendency to take charge) were distinguishing facets in addition to lower positive emotions (41).

##### Openness to Experience

Findings with respect to openness to experience in late-life suicide are inconsistent across studies comparing older suicide victims either to healthy controls or to older attempters. In comparison with healthy controls, Duberstein and colleagues initially found lower levels of openness to experience in older adults who died by suicide, with significant differences in the openness to esthetics (sensitivity to any form of art), and in the openness to action facets (preference of variety to routine) (41). These findings were, however, not replicated in further studies (when considering adjusted odds ratios in Tsoh and colleagues’ study) (49, 60). Compared to attempters, elderly victims were found to have either lower or not significantly different levels of openness to experience (49, 55). The effect of age was more consistent, with older suicide victims displaying lower levels of openness than their younger counterparts (41, 60, 63). Duberstein and colleagues identified that the greatest difference between the two age groups was in openness to action, indicating a higher preference for routine in older versus younger suicide victims (41).

Elderly suicide attempters showed no difference in openness to experience compared to healthy as well as depressed controls (46, 48, 49, 62).

In older suicide ideators, some (43, 51) but not all studies (48) found higher openness to experience when compared to depressed controls. No difference was found between ideators and healthy controls (59).

Thus, the role of openness to experience in late-life suicide remains unclear. According to Duberstein, lower levels of openness to experience in older suicide victims (compared to younger victims and healthy controls) may arise from a reduced capability to cope with age-related changes or losses due to excessive, short-sighted, concrete thinking and a rigidly defined self-image (111). Consistent with this theory, higher levels of openness to experience have been positively associated with volunteer work and better cognition in old age (10). It is, however, also possible that the lower levels of openness to action found in older versus younger adults who died by suicide may arise from a maturational effect, since a decrease in this facet has been associated with normal aging (112).

##### Conscientiousness

Higher levels of conscientiousness distinguished older suicide victims from younger victims (60, 63), as well as from older attempters in clinical samples (49, 55). The difference with attempters was also apparent at the facet level, where suicide victims displayed higher dutifulness (proneness to follow the rules), achievement-striving (tendency to work-hard/overachieve), and self-discipline (capability to get things done when needed) (55). Of three studies (41, 49, 60), only Tsoh and colleagues found lower levels conscientiousness in elderly suicide victims compared to general population controls in a univariate analysis, but the finding did not survive controlling for current major depression, past suicide attempts, physical comorbidities, sociodemographic factors and life events (49).

Studies comparing elderly suicide attempters versus general population controls found either lower conscientiousness in attempters (49) or similar levels in the two groups (61). No differences were found between older suicide attempters and depressed controls (43, 48). In a sex-stratified study, lower levels of conscientiousness were present in elderly male attempters compared to healthy controls, although no such difference was found in females (62).

Elderly suicide ideators showed no difference in their levels of conscientiousness compared to healthy or depressed controls (48, 51, 59).

Overall, higher levels of conscientiousness seem to discriminate suicides from unsuccessful attempts in depressed older adults, as well as late-onset suicides from early-onset ones. These relatively higher levels of conscientiousness in older suicides appear to be consistent with ICD/DSM-findings reporting an association with OCPD (see in the corresponding subsection). In a meta-analysis, OCPD was the only Axis-II diagnosis positively correlated with conscientiousness (113). Although higher levels of conscientiousness have been linked to several facets of successful aging (10, 114), the above findings suggest that this trait could also contribute to negative outcomes in late life, especially when co-occurring with depression.

##### Agreeableness

Studies have found no difference in agreeableness in older suicide victims compared to younger victims, healthy controls, and elderly attempters (41, 49, 55, 60, 63). Although Tsoh and colleagues found that elderly who died by suicide had higher levels of agreeableness than elderly attempters, this effect did not hold in the multivariate model controlling for current depressive disorder, history of suicide attempt, physical comorbidities, demographic factors, and life events (49).

No study found overall differences in agreeableness between elderly suicide attempters and healthy or depressed controls, with the exception of the unadjusted effect reported by Tsoh and colleagues that indicated lower levels of agreeableness in attempters (43, 49, 61, 62).

With respect to elderly suicide ideators, most studies found no difference in agreeableness compared to both healthy and depressed controls (48, 51, 59).

Thus, agreeableness does not seem to distinguish older adults who attempt or die by suicide from the general elderly population.

## Discussion

### Main Findings

Maladaptive personality seems to play a role in suicidal behavior in old age, although it is most often present in milder, subthreshold forms. The existing literature points toward heterogeneity in personality profiles of older adults with suicidal ideation or behavior. Suicidal ideation was predicted by narcissistic and borderline personality and was positively associated with all other personality disorders as indicated by at least one study, with the exception of histrionic personality. By contrast, only obsessive–compulsive and avoidant personality disorders were implicated in death by suicide, although this association may be confounded by depression.

Studies using the Big Five further characterized older adults who died by suicide as being more neurotic than healthy individuals, but less neurotic and more conscientious than older adults who only carried out non-fatal attempts. The limited evidence that we have links maladaptive personality with recurrent suicide attempts of largely lower severity. For example, lower anger–hostility was associated with a smaller number of attempts, higher lethality of method and higher intent to die, while lower positive emotions were identified as an independent predictor of a greater number of attempts (46).

We take these findings as evidence that a subset of aging individuals with personality disorders, BPD being the prototype, continue to display chronic suicidal ideation and repeated suicide attempts. On the other hand, the emergence of serious suicidal behavior in late life is associated with more intact personality in a majority of individuals and, possibly, obsessive–compulsive traits in a significant minority.

An important alternative hypothesis is that dysfunctional personality traits involved in late-life suicide are not well captured by existing criteria and measures, possibly reflecting effects of neurodegeneration and other aspects of brain aging. Cognitive rigidity, for instance, often appears in qualitative case descriptions and may sensitize older adults to acute stressors (56, 104, 105). It is, however, only partially captured by categorical OCPD criteria, higher conscientiousness, and lower openness to action (a facet of openness to experience), which may be overrepresented among late-life vs. early-life suicides.

### Limitations

The assessment of DSM/ICD personality disorder criteria may not be best suited for the characterization of personality in the elderly. DSM personality disorders map better onto younger than older adults in whom many of the criteria become difficult to evaluate (115, 116). In addition, personality assessment in psychological autopsy studies likely under-detects internalizing traits over the life course, reflected in only moderate consistency with ante-mortem Axis-II diagnoses found in adults (kappa = 0.65) (117). Therefore, older suicide attempters with highly medically lethal attempts probably provide the best *in vivo* window into death by suicide. Unfortunately, categorical data focusing on separate personality disorders in the elderly were absent in attempters, scarce in older suicide victims, and lacking comparisons with depressed non-suicidal control groups. These limitations make it impossible to draw strong conclusions about late-life suicidal behavior in patients with specific personality disorders.

Moreover, suicidal behavior is heterogeneous in its life course, planning, method selection, and medical consequences (106, 118). The studies reviewed here did not generally consider this heterogeneity in their design and analytic approach, and the relationship between the older individual’s personality profile and the life course and characteristics of his/her suicidal behavior remains largely unexplored. More work is needed, both to identify reliable predictors of any suicidal behavior in old age and, more importantly, parse the behavior’s heterogeneity.

### Clinical Implications

From a clinical perspective, these results suggest that older adults who are evaluated for suicidal ideation are not necessarily representative of those who die by suicide. Among the latter individuals, only a subgroup seems to display dysfunctional personality features, with a rigid vulnerability being possibly as common as an emotionally labile one. This heterogeneity highlights the challenges in assessing suicidal risk in the elderly. One needs to be cautious in drawing conclusions from the patient’s personality profile, and other risk factors such as access to firearms, depression, addiction, psychosis, and pain should be given greater weight at the current state of knowledge.

### Future Directions

Disorders of aging can also alter personality. Dementia brings about a progressive personality change (119), exaggeration of existing personality traits and mood disturbances being the most frequent manifestations. A longitudinal study found that, in prodromal Alzheimer’s disease, increased rigidity was the most frequent alteration, occurring in 25% of the individuals subsequently converting to dementia, closely followed by apathy (24%), egocentricity (21%), and impaired emotional control (18%) (120). Other investigators reported higher levels of neuroticism, as well as lower levels of both extraversion and conscientiousness in patients with Alzheimer type dementia (121, 122). Similarly, higher levels of neuroticism, as well as lower levels of extraversion and goal orientation have been reported in either premorbid or beginning stages of Parkinson’s disease (123–125). Such personality characteristics may be associated with alterations in the dopaminergic system and therefore predate motor symptoms of parkinsonism (126). Thus, the behavioral effects of neurodegeneration may in part explain the divergence in personality profiles between younger and older suicides. For example, it is possible that the accentuation of rigid personality traits contributes to suicide risk by impairing individuals’ ability to cope with their progressive loss of cognitive abilities. Moreover, organic mental disorder may similarly contribute to the heterogeneity of personality profiles found in older adults at risk of suicide and has been linked to an overall under-detection of personality disorders in the elderly (77). Interestingly, new forms of impulsivity may also emerge in neurocognitive disorders, as in the case of the disruption of the indirect (no-go) and hyper-direct (“cortical brake”) pathways by dopamine agonists and subthalamic nucleus stimulation in Parkinson’s disease, where some studies point to treatment-emergent suicidal behavior (127, 128).

Another neurocognitive mechanism worth investigating further is executive dysfunction. This construct has already been linked to late-onset, treatment-resistant depression (129, 130) as well as to suicidal behavior (131). Moreover, there appears to be a correlation between deficits in executive functioning and certain personality disorders, such as antisocial, borderline and obsessive–compulsive (132–134).

The question of the best approach to characterize personality in older adults at increased risk of suicide remains open. Research is increasingly shifting toward dimensional measures, which have the advantages of being assessable through self-report and increasing statistical power by virtue of being parametric. Clinical constructs underlying categorical personality disorders, however, appear to possess unique explanatory and heuristic value above and beyond the Big Five in the study of suicidal behavior (135). In addition to traditional personality measures, complementary neuropsychological tools may be needed, including experimental paradigms assessing decision-making and social interactions, similarly to the way other trait-level constructs, such as impulsivity, are investigated (136).

### Conclusion

Despite the scarcity of published studies, this review highlights (1) the smaller role that Cluster B personality disorders and their defining constructs play in late-life suicide compared to suicide in younger adults and (2) a possible role for Cluster C disorders, such as OCPD, in late-life suicide. Almost nothing is known about the contribution of personality change due to neurodegenerative and vascular diseases. The reasons for the generally lower prevalence of diagnosable personality disorders in older vs. younger adults who engage in suicidal behavior are likely to include actual change in the behavioral expression of personality across the lifespan, poorer fit of diagnostic instruments, and the contributions of neurodegenerative and vascular brain changes. We would be remiss not to mention the possibility, often rejected by psychiatrists, that some proportion of late-life suicidal behavior may be rational in that it is not explained by psychopathology. Future research will reveal to what extent this may be true, and to what extent suicide in characterologically unaffected older adults is motivated by concerns such as depression, psychosis, addiction, or impaired cognition and decision-making.

## Author Contributions

AD and KS conceived and directed the project; AS collected data, structured and drafted the article. All authors contributed to the theoretical framework, data interpretation as well as the development of the manuscript.

## Conflict of Interest Statement

The authors declare that the research was conducted in the absence of any commercial or financial relationships that could be construed as a potential conflict of interest.

## References

[1] National Institute of Mental Health (NIMH). Suicide [Internet]. (2017) [cited 2018 Feb 27]. Available from: https://www.nimh.nih.gov/health/statistics/suicide.shtml#part_154969

[2] American Association of Suicidology. Elderly Suicide Fact Sheet Based on 2012 Data [Internet]. (2014) [cited 2016 Dec 19]. Available from: http://www.suicidology.org/Portals/14/docs/Resources/FactSheets/Elderly2012.pdf

[3] Waern M Rubenowitz E Runeson B Skoog I Wilhelmson K Allebeck P. Burden of illness and suicide in elderly people: case-control study. BMJ (2002) 324(7350):1355. 10.1136/bmj.324.7350.1355 12052799 PMC115206

[4] Waern M Rubenowitz E Wilhelmson K. Predictors of suicide in the old elderly. Gerontology (2003) 49(5):328–34. 10.1159/000071715 12920354

[5] Juurlink DN Herrmann N Szalai JP Kopp A Redelmeier DA. Medical illness and the risk of suicide in the elderly. Arch Intern Med (2004) 164(11):1179–84. 10.1001/archinte.164.11.1179 15197042

[6] Fässberg MM Cheung G Canetto SS Erlangsen A Lapierre S Lindner R A systematic review of physical illness, functional disability, and suicidal behaviour among older adults. Aging Ment Health (2016) 20(2):166–94. 10.1080/13607863.2015.1083945 26381843 PMC4720055

[7] Mann JJ Waternaux C Haas GL Malone KM. Toward a clinical model of suicidal behavior in psychiatric patients. Am J Psychiatry (1999) 156(2):181–9. 9989552 10.1176/ajp.156.2.181

[8] Heikkinen ME Henriksson MM Isometsä ET Marttunen MJ Na HM LöNnqvist JK. Recent life events and suicide in personality disorders. J Nerv Amp Ment Dis (1997) 185(6):373–81. 10.1097/00005053-199706000-00003 9205423

[9] Devanand D. Comorbid psychiatric disorders in late life depression. Biol Psychiatry (2002) 52(3):236–42. 10.1016/S0006-3223(02)01336-7 12182929

[10] Baek Y Martin P Siegler IC Davey A Poon LW. Personality traits and successful aging: findings from the Georgia centenarian study. Int J Aging Hum Dev (2016) 83(3):207–27. 10.1177/0091415016652404 27298487

[11] Medawar PB Uniqueness of the Individual. London: Methuen (1957).

[12] Williams GC Williams DC Natural selection of individually harmful social adaptations among sibs with special reference to social insects. Evolution (1957) 11(1):32–9. 10.2307/2405809

[13] Giner L Blasco-Fontecilla H Mercedes Perez-Rodriguez M Garcia-Nieto R Giner J Guija JA Personality disorders and health problems distinguish suicide attempters from completers in a direct comparison. J Affect Disord (2013) 151(2):474–83. 10.1016/j.jad.2013.06.029 23859005

[14] May AM Klonsky ED Klein DN. Predicting future suicide attempts among depressed suicide ideators: a 10-year longitudinal study. J Psychiatr Res (2012) 46(7):946–52. 10.1016/j.jpsychires.2012.04.009 22575331 PMC3372684

[15] Ansell EB Wright AGC Markowitz JC Sanislow CA Hopwood CJ Zanarini MC Personality disorder risk factors for suicide attempts over 10 years of follow-up. Personal Disord (2015) 6(2):161–7. 10.1037/per0000089 25705977 PMC4415153

[16] Diaconu G Turecki G. Obsessive-compulsive personality disorder and suicidal behavior: evidence for a positive association in a sample of depressed patients. J Clin Psychiatry (2009) 70(11):1551–6. 10.4088/JCP.08m04636 19607764

[17] Verona E Sachs-Ericsson N Joiner TE. Suicide attempts associated with externalizing psychopathology in an epidemiological sample. Am J Psychiatry (2004) 161(3):444–51. 10.1176/appi.ajp.161.3.444 14992969

[18] Foster T Gillespie K McClelland R Patterson C Risk factors for suicide independent of DSM-III-R axis I disorder. Case-control psychological autopsy study in northern Ireland. Br J Psychiatry (1999) 175(2):175–9. 10.1192/bjp.175.2.175 10627802

[19] Brezo J Paris J Turecki G. Personality traits as correlates of suicidal ideation, suicide attempts, and suicide completions: a systematic review. Acta Psychiatr Scand (2006) 113(3):180–206. 10.1111/j.1600-0447.2005.00702.x 16466403

[20] Fang L Heisel MJ Duberstein PR Zhang J. Combined effects of neuroticism and extraversion: findings from a matched case control study of suicide in rural China. J Nerv Ment Dis (2012) 200(7):598–602. 10.1097/NMD.0b013e31825bfb53 22759937

[21] Pallis DJ Jenkins JS Extraversion, neuroticism, and intent in attempted suicides. Psychol Rep (1977) 41(1):19–22. 10.2466/pr0.1977.41.1.19 909989

[22] Stanković Z Saula-Marojević B Potrebić A. Personality profile of depressive patients with a history of suicide attempts. Psychiatr Danub (2006) 18(3–4):159–68. 17099606

[23] Yen S Siegler IC Self-blame, social introversion, and male suicides: prospective data from a longitudinal study. Arch Suicide Res (2003) 7(1):17–27. 10.1080/13811110301569

[24] Brodsky BS Groves SA Oquendo MA Mann JJ Stanley B. Interpersonal precipitants and suicide attempts in borderline personality disorder. Suicide Life Threat Behav (2006) 36(3):313–22. 10.1521/suli.2006.36.3.313 16805659

[25] Rihmer Z Benazzi F. Impact on suicidality of the borderline personality traits impulsivity and affective instability. Ann Clin Psychiatry (2010) 22(2):121–8. 20445839

[26] Stone MH Hurt SW Stone DK The PI 500: long-term follow-up of borderline inpatients meeting DSM-III criteria I. Global outcome. J Personal Disord (1987) 1(4):291–8. 10.1521/pedi.1987.1.4.291

[27] Zanarini MC Frankenburg FR Reich DB Fitzmaurice G. Time to attainment of recovery from borderline personality disorder and stability of recovery: a 10-year prospective follow-up study. Am J Psychiatry (2010) 167(6):663–7. 10.1176/appi.ajp.2009.09081130 20395399 PMC3203735

[28] Frierson RL. Suicide attempts by the old and the very old. Arch Intern Med (1991) 151(1):141. 10.1001/archinte.151.1.141 1985589

[29] Conwell Y Duberstein PR Cox C Herrmann J Forbes N Caine ED. Age differences in behaviors leading to completed suicide. Am J Geriatr Psychiatry (1998) 6(2):122–6. 10.1097/00019442-199805000-00005 9581207

[30] American Psychological Association (APA). Personality [Internet]. (2018) [cited 2018 Feb 6]. Available from: http://www.apa.org/topics/personality/index.aspx

[31] O’Carroll PW Berman AL Maris RW Moscicki EK Tanney BL Silverman MM. Beyond the tower of Babel: a nomenclature for suicidology. Suicide Life Threat Behav (1996) 26(3):237–52. 8897663

[32] Chan LF Shamsul AS Maniam T. Are predictors of future suicide attempts and the transition from suicidal ideation to suicide attempts shared or distinct: a 12-month prospective study among patients with depressive disorders. Psychiatry Res (2014) 220(3):867–73. 10.1016/j.psychres.2014.08.055 25240940

[33] Suominen K Isometsä E Suokas J Haukka J Achte K Lönnqvist J. Completed suicide after a suicide attempt: a 37-year follow-up study. Am J Psychiatry (2004) 161(3):562–3. 10.1176/appi.ajp.161.3.562 14992984

[34] Rudd MD Joiner T Rajab MH. Relationships among suicide ideators, attempters, and multiple attempters in a young-adult sample. J Abnorm Psychol (1996) 105(4):541–50. 10.1037/0021-843X.105.4.541 8952187

[35] Gil S. Suicide attempters vs. ideators: are there differences in personality profiles? Arch Suicide Res (2005) 9(2):153–61. 10.1080/13811110590904007 16020159

[36] Brezo J Paris J Tremblay R Vitaro F HéBert M Turecki G. Identifying correlates of suicide attempts in suicidal ideators: a population-based study. Psychol Med (2007) 37(11):1551–62. 10.1017/S0033291707000803 17537281

[37] Ciompi L Late suicide in former mental patients. Psychopathology (1976) 9(1):59–63. 10.1159/000283665 1019369

[38] Nieto E Vieta E Lazaro L Gasto C Cirera E. Serious suicide attempts in the elderly. Psychopathology (1992) 25(4):183–8. 10.1159/000284770 1492141

[39] Kunik ME Mulsant BH Rifai AH Sweet R Pasternak R Rosen J Personality disorders in elderly inpatients with major depression. Am J Geriatr Psychiatry (1993) 1(1):38–45. 10.1097/00019442-199300110-00006 28530944

[40] Draper B Suicidal behaviour in the elderly. Int J Geriatr Psychiatry (1994) 9(8):655–61. 10.1002/gps.930090810

[41] Duberstein PR Conwell Y Caine ED Age differences in the personality characteristics of suicide completers: preliminary findings from a psychological autopsy study. Psychiatry (1994) 57(3):213–24. 10.1080/00332747.1994.11024686 7800770

[42] Henriksson MM Marttunen MJ Isometsä ET Heikkinen ME Aro HM Kuoppasalmi KI Mental disorders in elderly suicide. Int Psychogeriatr (1995) 7(2):275–86. 10.1017/S1041610295002031 8829433

[43] Duberstein PR Conwell Y Seidlitz L Denning DG Cox C Caine ED Personality traits and suicidal behavior and ideation in depressed inpatients 50 years of age and older. J Gerontol B Psychol Sci Soc Sci (2000) 55(1):18–26. 10.1093/geronb/55.1.P18 10728121

[44] Lawrence D Almeida OP Hulse GK Jablensky AV Holman CD. Suicide and attempted suicide among older adults in Western Australia. Psychol Med (2000) 30(4):813–21. 10.1017/S0033291799002391 11037089

[45] Harwood D Hawton K Hope T Jacoby R. Psychiatric disorder and personality factors associated with suicide in older people: a descriptive and case-control study. Int J Geriatr Psychiatry (2001) 16(2):155–65. 10.1002/1099-1166(200102)16:2<155::AID-GPS289>3.0.CO;2-0 11241720

[46] Seidlitz L Conwell Y Duberstein P Cox C Denning D. Emotion traits in older suicide attempters and non-attempters. J Affect Disord (2001) 66(2–3):123–31. 10.1016/S0165-0327(00)00300-1 11578664

[47] Morse JQ Lynch TR. A preliminary investigation of self-reported personality disorders in late life: prevalence, predictors of depressive severity, and clinical correlates. Aging Ment Health (2004) 8(4):307–15. 10.1080/13607860410001709674 15370047

[48] Useda JD Duberstein PR Conner KR Conwell Y. Personality and attempted suicide in depressed adults 50 years of age and older: a facet level analysis. Compr Psychiatry (2004) 45(5):353–61. 10.1016/j.comppsych.2004.06.002 15332198

[49] Tsoh J Chiu HFK Duberstein PR Chan SSM Chi I Yip PSF Attempted suicide in elderly Chinese persons: a multi-group, controlled study. Am J Geriatr Psychiatry (2005) 13(7):562–71. 10.1097/00019442-200507000-00004 16009732

[50] Harwood D Hawton K Hope T Jacoby R. Suicide in older people without psychiatric disorder. Int J Geriatr Psychiatry (2006) 21(4):363–7. 10.1002/gps.1473 16534772

[51] Heisel MJ Duberstein PR Conner KR Franus N Beckman A Conwell Y. Personality and reports of suicide ideation among depressed adults 50 years of age or older. J Affect Disord (2006) 90(2–3):175–80. 10.1016/j.jad.2005.11.005 16380165

[52] Hunt IM Kapur N Robinson J Shaw J Flynn S Bailey H Suicide within 12 months of mental health service contact in different age and diagnostic groups: national clinical survey. Br J Psychiatry (2006) 188(2):135–42. 10.1192/bjp.188.2.135 16449700

[53] Heisel MJ Links PS Conn D van Reekum R Flett GL. Narcissistic personality and vulnerability to late-life suicidality. Am J Geriatr Psychiatry (2007) 15(9):734–41. 10.1097/01.JGP.0000260853.63533.7d 17804827

[54] Hirsch JK Duberstein PR Chapman B Lyness JM. Positive affect and suicide ideation in older adult primary care patients. Psychol Aging (2007) 22(2):380–5. 10.1037/0882-7974.22.2.380 17563193 PMC4846281

[55] Useda JD Duberstein PR Conner KR Beckman A Franus N Tu X Personality differences in attempted suicide versus suicide in adults 50 years of age or older. J Consult Clin Psychol (2007) 75(1):126–33. 10.1037/0022-006X.75.1.126 17295571

[56] Kjølseth I Ekeberg O Steihaug S “Why do they become vulnerable when faced with the challenges of old age?” Elderly people who committed suicide, described by those who knew them. Int Psychogeriatr (2009) 21(5):903–12. 10.1017/S1041610209990342 19519985

[57] Miret M Nuevo R Morant C Sainz-Cortón E Jiménez-Arriero MA López-Ibor JJ Differences between younger and older adults in the structure of suicidal intent and its correlates. Am J Geriatr Psychiatry (2010) 18(9):839–47. 10.1097/JGP.0b013e3181d145b0 20220600

[58] Qin P. The impact of psychiatric illness on suicide: differences by diagnosis of disorders and by sex and age of subjects. J Psychiatr Res (2011) 45(11):1445–52. 10.1016/j.jpsychires.2011.06.002 21722920

[59] Segal DL Marty MA Meyer WJ Coolidge FL. Personality, suicidal ideation, and reasons for living among older adults. J Gerontol B Psychol Sci Soc Sci (2012) 67(2):159–66. 10.1093/geronb/gbr080 21765174

[60] De Leo D Draper BM Snowdon J Kõlves K Suicides in older adults: a case–control psychological autopsy study in Australia. J Psychiatr Res (2013) 47(7):980–8. 10.1016/j.jpsychires.2013.02.009 23522934

[61] Wiktorsson S Berg AI Billstedt E Duberstein PR Marlow T Skoog I Neuroticism and extroversion in suicide attempters aged 75 and above and a general population comparison group. Aging Ment Health (2013) 17(4):479–88. 10.1080/13607863.2012.749835 23336286

[62] Chan SMS Chiu FKH Lam CWL Wong SMC Conwell Y. A multidimensional risk factor model for suicide attempts in later life. Neuropsychiatr Dis Treat (2014) 10:1807–17. 10.2147/NDT.S70011 25258538 PMC4174030

[63] Draper B Kõlves K De Leo D Snowdon J A controlled study of suicide in middle-aged and older people: personality traits, age, and psychiatric disorders. Suicide Life Threat Behav (2014) 44(2):130–8. 10.1111/sltb.12053 23952907

[64] Kim BJ Ahn J. Factors that influence suicidal ideation among elderly Korean immigrants: focus on diatheses and stressors. Aging Ment Health (2014) 18(5):619–27. 10.1080/13607863.2013.866631 24328389

[65] Jahn DR Poindexter EK Cukrowicz KC. Personality disorder traits, risk factors, and suicide ideation among older adults. Int Psychogeriatr (2015) 27(11):1785–94. 10.1017/S1041610215000174 25706934

[66] Segal DL Gottschling J Marty M Meyer WJ Coolidge FL. Relationships among depressive, passive-aggressive, sadistic and self-defeating personality disorder features with suicidal ideation and reasons for living among older adults. Aging Ment Health (2015) 19(12):1071–7. 10.1080/13607863.2014.1003280 25621821

[67] Eades A Segal DL Coolidge F Feliciano L Suicide Among Older Adults: An Exploration of the Effects of Personality and Self-Esteem on Thwarted Belongingness, Perceived Burdensomeness, and suicidal ideation [MA Thesis]. Colorado Springs: University of Colorado, Department of Psychology (2016).

[68] Costa PT McCrae RR Primary traits of Eysenck’s P-E-N system: three- and five-factor solutions. J Pers Soc Psychol (1995) 69(2):308–17. 10.1037/0022-3514.69.2.308 7643307

[69] Saucier G. Replicable item-cluster subcomponents in the NEO five-factor inventory. J Pers Assess (1998) 70(2):263–76. 10.1207/s15327752jpa7002_6 9697330

[70] Multisite Intervention Study on Suicidal Behaviours-SUPRE-MISS: Protocol of SUPRE-MISS. World Health Organization – Multisite Intervention Study on Suicidal Behaviours 2002.

[71] Mościcki EK. Gender differences in completed and attempted suicides. Ann Epidemiol (1994) 4(2):152–8. 10.1016/1047-2797(94)90062-0 8205283

[72] Waern M Runeson BS Allebeck P Beskow J Rubenowitz E Skoog I Mental disorder in elderly suicides: a case-control study. Am J Psychiatry (2002) 159(3):450–5. 10.1176/appi.ajp.159.3.450 11870010

[73] Devanand DP Turret N Moody BJ Fitzsimons L Peyser S Mickle K Personality disorders in elderly patients with dysthymic disorder. Am J Geriatr Psychiatry (2000) 8(3):188–95. 10.1097/00019442-200008000-00002 10910415

[74] Harrison KE Dombrovski AY Morse JQ Houck P Schlernitzauer M Reynolds CF Alone? Perceived social support and chronic interpersonal difficulties in suicidal elders. Int Psychogeriatr (2010) 22(03):445 10.1017/S1041610209991463 20003633 PMC3045785

[75] Waern M Beskow J Runeson B Skoog I. Suicidal feelings in the last year of life in elderly people who commit suicide. The Lancet (1999) 354(9182):917–8. 10.1016/S0140-6736(99)93099-4 10489955

[76] Abrams RC Horowitz SV Personality disorders after age 50: a meta-analysis. J Personal Disord (1996) 10(3):271–81. 10.1521/pedi.1996.10.3.271

[77] Kunik ME Mulsant BH Rifai AH Sweet R Pasternak R Zubenko GS. Diagnostic rate of comorbid personality disorder in elderly psychiatric inpatients. Am J Psychiatry (1994) 151(4):603–5. 10.1176/ajp.151.4.603 8147462

[78] Koenig HG Meador KG Cohen HJ Blazer DG Detection and treatment of major depression in older medically ill hospitalized patients. Int J Psychiatry Med (1989) 18(1):17–31. 10.2190/QUP8-XL19-TKXK-CRE5 3397224

[79] German PS Shapiro S Skinner EA Von Korff M Klein LE Turner RW Detection and management of mental health problems of older patients by primary care providers. JAMA (1987) 257(4):489. 10.1001/jama.257.4.489 3540329

[80] Uncapher H Areán PA. Physicians are less willing to treat suicidal ideation in older patients. J Am Geriatr Soc (2000) 48(2):188–92. 10.1111/j.1532-5415.2000.tb03910.x 10682948

[81] Magnavita JJ Handbook of Personality Disorders – Theory and Practice [Internet]. New Jersey, United States: John Wiley & Sons, Inc (2004).

[82] Grant BF Hasin DS Stinson FS Dawson DA Chou SP Ruan WJ Prevalence, correlates, and disability of personality disorders in the United States: results from the national epidemiologic survey on alcohol and related conditions. J Clin Psychiatry (2004) 65(7):948–58. 10.4088/JCP.v65n0711 15291684

[83] Torgersen S Kringlen E Cramer V. The prevalence of personality disorders in a community sample. Arch Gen Psychiatry (2001) 58(6):590–6. 10.1001/archpsyc.58.6.590 11386989

[84] Engels GI Duijsens IJ Haringsma R van Putten CM. Personality disorders in the elderly compared to four younger age groups: a cross-sectional study of community residents and mental health patients. J Pers Disord (2003) 17(5):447–59. 10.1521/pedi.17.5.447.22971 14632377

[85] Zimmerman M Coryell W. DSM-III personality disorder diagnoses in a nonpatient sample: demographic correlates and comorbidity. Arch Gen Psychiatry (1989) 46(8):682. 10.1001/archpsyc.1989.01810080012002 2751402

[86] Lentz V Robinson J Bolton JM. Childhood adversity, mental disorder comorbidity, and suicidal behavior in schizotypal personality disorder. J Nerv Ment Dis (2010) 198(11):795–801. 10.1097/NMD.0b013e3181f9804c 21048469

[87] Soloff PH Lis JA Kelly T Cornelius J Ulrich R. Risk factors for suicidal behavior in borderline personality disorder. Am J Psychiatry (1994) 151(9):1316–23. 10.1176/ajp.151.9.1316 8067487

[88] Roth KB Borges G Medina-Mora M-E Orozco R Ouéda C Wilcox HC. Depressed mood and antisocial behavior problems as correlates for suicide-related behaviors in Mexico. J Psychiatr Res (2011) 45(5):596–602. 10.1016/j.jpsychires.2010.10.009 21055767

[89] Black DW Baumgard CH Bell SE. A 16- to 45-year follow-up of 71 men with antisocial personality disorder. Compr Psychiatry (1995) 36(2):130–40. 10.1016/S0010-440X(95)90108-6 7758299

[90] Zanarini MC Frankenburg FR Reich DB Fitzmaurice GM. Fluidity of the subsyndromal phenomenology of borderline personality disorder over 16 years of prospective follow-up. Am J Psychiatry (2016) 173(7):688–94. 10.1176/appi.ajp.2015.15081045 26869248 PMC4930411

[91] Paris J Zweig-Frank H. A 27-year follow-up of patients with borderline personality disorder. Compr Psychiatry (2001) 42(6):482–7. 10.1053/comp.2001.26271 11704940

[92] Pompili M Girardi P Ruberto A Tatarelli R. Suicide in borderline personality disorder: a meta-analysis. Nord J Psychiatry (2005) 59(5):319–24. 10.1080/08039480500320025 16757458

[93] Shearer SL Peters CP Quaytman MS Wadman BE. Intent and lethality of suicide attempts among female borderline inpatients. Am J Psychiatry (1988) 145(11):1424–7. 10.1176/ajp.145.11.1424 3189601

[94] Kernberg OF Internal World and External Reality. New York, United States: Jason Aronson, Inc (1980).

[95] McGlashan TH Grilo CM Sanislow CA Ralevski E Morey LC Gunderson JG Two-year prevalence and stability of individual DSM-IV criteria for schizotypal, borderline, avoidant, and obsessive-compulsive personality disorders: toward a hybrid model of axis II disorders. Am J Psychiatry (2005) 162(5):883–9. 10.1176/appi.ajp.162.5.883 15863789 PMC3272783

[96] Alexopoulos GS Depression in the elderly. The Lancet (2005) 365(9475):1961–70. 10.1016/S0140-6736(05)66665-2 15936426

[97] Yen S Pagano ME Shea MT Grilo CM Gunderson JG Skodol AE Recent life events preceding suicide attempts in a personality disorder sample: findings from the collaborative longitudinal personality disorders study. J Consult Clin Psychol (2005) 73(1):99–105. 10.1037/0022-006X.73.1.99 15709836 PMC3276403

[98] Diedrich A Voderholzer U. Obsessive–compulsive personality disorder: a current review. Curr Psychiatry Rep (2015) 17(2):2. 10.1007/s11920-014-0547-8 25617042

[99] Grant JE Mooney ME Kushner MG. Prevalence, correlates, and comorbidity of DSM-IV obsessive-compulsive personality disorder: results from the national epidemiologic survey on alcohol and related conditions. J Psychiatr Res (2012) 46(4):469–75. 10.1016/j.jpsychires.2012.01.009 22257387

[100] Ullrich S Coid J. The age distribution of self-reported personality disorder traits in a household population. J Pers Disord (2009) 23(2):187–200. 10.1521/pedi.2009.23.2.187 19379095

[101] Chen H Cohen P Crawford TN Kasen S Johnson JG Berenson K. Relative impact of young adult personality disorders on subsequent quality of life: findings of a community-based longitudinal study. J Pers Disord (2006) 20(5):510–23. 10.1521/pedi.2006.20.5.510 17032162

[102] Skodol AE Gunderson JG McGlashan TH Dyck IR Stout RL Bender DS Functional impairment in patients with schizotypal, borderline, avoidant, or obsessive-compulsive personality disorder. Am J Psychiatry (2002) 159(2):276–83. 10.1176/appi.ajp.159.2.276 11823271

[103] Grilo CM Skodol AE Gunderson JG Sanislow CA Stout RL Shea MT Longitudinal diagnostic efficiency of DSM-IV criteria for obsessive-compulsive personality disorder: a 2-year prospective study. Acta Psychiatr Scand (2004) 110(1):64–8. 10.1046/j.0001-690X.2003.00223.x 15180781

[104] Conwell Y Caine ED Olsen K. Suicide and cancer in late life. Hosp Community Psychiatry (1990) 41(12):1334–9. 2276727 10.1176/ps.41.12.1334

[105] Horton-Deutsch SL Clark DC Farran CJ. Chronic dyspnea and suicide in elderly men. Hosp Community Psychiatry (1992) 43(12):1198–203. 1459540 10.1176/ps.43.12.1198

[106] Dombrovski AY Szanto K Siegle GJ Wallace ML Forman SD Sahakian B Lethal forethought: delayed reward discounting differentiates high- and low-lethality suicide attempts in old age. Biol Psychiatry (2011) 70(2):138–44. 10.1016/j.biopsych.2010.12.025 21329911 PMC3125431

[107] Pinto A Steinglass JE Greene AL Weber EU Simpson HB. Capacity to delay reward differentiates obsessive-compulsive disorder and obsessive-compulsive personality disorder. Biol Psychiatry (2014) 75(8):653–9. 10.1016/j.biopsych.2013.09.007 24199665 PMC3969772

[108] Dirks BL Repetition of parasuicide – ICD-10 personality disorders and adversity. Acta Psychiatr Scand (1998) 98(3):208–13. 10.1111/j.1600-0447.1998.tb10068.x 9761407

[109] Raja M Azzoni A. The impact of obsessive-compulsive personality disorder on the suicidal risk of patients with mood disorders. Psychopathology (2007) 40(3):184–90. 10.1159/000100366 17337939

[110] Wilson RS Krueger KR Gu L Bienias JL Mendes de Leon CF Evans DA. Neuroticism, extraversion, and mortality in a defined population of older persons. Psychosom Med (2005) 67(6):841–5. 10.1097/01.psy.0000190615.20656.83 16314587

[111] Duberstein PR. Openness to experience and completed suicide across the second half of life. Int Psychogeriatr (1995) 7(2):183–98. 10.1017/S1041610295001967 8829426

[112] Costa PT McCrae RR. Personality in adulthood: a six-year longitudinal study of self-reports and spouse ratings on the NEO personality inventory. J Pers Soc Psychol (1988) 54(5):853–63. 10.1037/0022-3514.54.5.853 3379583

[113] Samuel D Widiger T A meta-analytic review of the relationships between the five-factor model and DSM-IV-TR personality disorders: a facet level analysis. Clin Psychol Rev (2008) 28(8):1326–42. 10.1016/j.cpr.2008.07.002 18708274 PMC2614445

[114] Wilson RS Boyle PA Yu L Segawa E Sytsma J Bennett DA. Conscientiousness, dementia related pathology, and trajectories of cognitive aging. Psychol Aging (2015) 30(1):74–82. 10.1037/pag0000013 25664558 PMC4361241

[115] Casey DA Schrodt CJ. Axis II diagnoses in geriatric inpatients. J Geriatr Psychiatry Neurol (1989) 2(2):87–8. 2775441 10.1177/089198878900200206

[116] Fogel BS Westlake R. Personality disorder diagnoses and age in inpatients with major depression. J Clin Psychiatry (1990) 51(6):232–5. 2347860

[117] Kelly TM Mann JJ. Validity of DSM-III-R diagnosis by psychological autopsy: a comparison with clinician ante-mortem diagnosis. Acta Psychiatr Scand (1996) 94(5):337–43. 10.1111/j.1600-0447.1996.tb09869.x 9124080

[118] Szanto K Galfalvy H Vanyukov PM Keilp JG Dombrovski AY Pathways to late-life suicidal behavior: cluster analysis and predictive validation of suicidal behavior. J Clin Psychiatry (2017) 79(2):17m11611 10.4088/JCP.17m11611 PMC5932247 29489076

[119] Helmes E Norton MC Østbye T Personality change in older adults with dementia: occurrence and association with severity of cognitive impairment. Adv Aging Res (2013) 2(1):27–36. 10.4236/aar.2013.21004

[120] Balsis S Carpenter BD Storandt M. Personality change precedes clinical diagnosis of dementia of the Alzheimer type. J Gerontol B Psychol Sci Soc Sci (2005) 60(2):98–101. 10.1093/geronb/60.2.P98 15746024

[121] Duchek JM Balota DA Storandt M Larsen R The power of personality in discriminating between healthy aging and early-stage Alzheimer’s disease. J Gerontol B Psychol Sci Soc Sci (2007) 62(6):353–61. 10.1093/geronb/62.6.P353 18079420

[122] Duberstein PR Chapman BP Tindle HA Sink KM Bamonti P Robbins J Personality and risk for Alzheimer’s disease in adults 72 years of age and older: a 6-year follow-up. Psychol Aging (2011) 26(2):351–62. 10.1037/a0023405 20973606 PMC3115437

[123] Bower JH Grossardt BR Maraganore DM Ahlskog JE Colligan RC Geda YE Anxious personality predicts an increased risk of Parkinson’s disease. Mov Disord (2010) 25(13):2105–13. 10.1002/mds.23230 20669309 PMC3089895

[124] Heberlein I Ludin H-P Scholz J Vieregge P Personality, depression, and premorbid lifestyle in twin pairs discordant for Parkinson’s disease. J Neurol Neurosurg Psychiatry (1998) 64(2):262–6. 10.1136/jnnp.64.2.262 9489545 PMC2169972

[125] Glosser G Clark C Freundlich B Kliner-Krenzel L Flaherty P Stern M A controlled investigation of current and premorbid personality: characteristics of Parkinson’s disease patients. Mov Disord (1995) 10(2):201–6. 10.1002/mds.870100211 7753062

[126] Menza MA Golbe LI Cody RA Forman NE Dopamine-related personality traits in Parkinson’s disease. Neurology (1993) 43(3 Pt 1):505–8. 10.1212/WNL.43.3_Part_1.505 8450991

[127] Voon V Krack P Lang AE Lozano AM Dujardin K Schüpbach M A multicentre study on suicide outcomes following subthalamic stimulation for Parkinson’s disease. Brain (2008) 131(10):2720 10.1093/brain/awn214 18941146 PMC2724899

[128] Lee T Lee HB Ahn MH Kim J Kim MS Chung SJ Increased suicide risk and clinical correlates of suicide among patients with Parkinson’s disease. Parkinsonism Relat Disord (2016) 32:102–7. 10.1016/j.parkreldis.2016.09.006 27637284

[129] Lockwood KA Alexopoulos GS van Gorp WG. Executive dysfunction in geriatric depression. Am J Psychiatry (2002) 159(7):1119–26. 10.1176/appi.ajp.159.7.1119 12091189

[130] Alexopoulos GS Kiosses DN Heo M Murphy CF Shanmugham B Gunning-Dixon F. Executive dysfunction and the course of geriatric depression. Biol Psychiatry (2005) 58(3):204–10. 10.1016/j.biopsych.2005.04.024 16018984

[131] Richard-Devantoy S Berlim MT Jollant F. A meta-analysis of neuropsychological markers of vulnerability to suicidal behavior in mood disorders. Psychol Med (2014) 44(8):1663–73. 10.1017/S0033291713002304 24016405

[132] LeGris J van Reekum R. The neuropsychological correlates of borderline personality disorder and suicidal behaviour. Can J Psychiatry (2006) 51(3):131–42. 10.1177/070674370605100303 16618004

[133] Krakowski MI Foxe J de Sanctis P Nolan K Hoptman MJ Shope C Aberrant response inhibition and task switching in psychopathic individuals. Psychiatry Res (2015) 229(3):1017–23. 10.1016/j.psychres.2015.06.018 26257091

[134] Fineberg NA Day GA de Koenigswarter N Reghunandanan S Kolli S Jefferies-Sewell K The neuropsychology of obsessive-compulsive personality disorder: a new analysis. CNS Spectr (2015) 20(5):490–9. 10.1017/S1092852914000662 25776273

[135] Morey LC Zanarini MC. Borderline personality: traits and disorder. J Abnorm Psychol (2000) 109(4):733–7. 10.1037/0021-843X.109.4.733 11195998

[136] Sharma L Markon KE Clark LA Toward a theory of distinct types of “impulsive” behaviors: a meta-analysis of self-report and behavioral measures. Psychol Bull (2014) 140(2):374–408. 10.1037/a0034418 24099400

